# Novel Vulgarin Derivatives: Chemical Transformation, In Silico and In Vitro Studies

**DOI:** 10.3390/molecules28083421

**Published:** 2023-04-13

**Authors:** Hanan G. Sary, Mohammed A. Khedr, Khaled Y. Orabi

**Affiliations:** 1 Department of Pharmaceutical Chemistry, College of Pharmacy, Kuwait University, Safat 13110, Kuwait; hanan.sary@ku.edu.kwmohammed.khedr@ku.edu.kw (M.A.K.); 2 Department of Pharmacognosy, Faculty of Pharmacy, Ain-Shams University, Cairo 11566, Egypt

**Keywords:** vulgarin, scaffold hopping, sigmatropic, chemical transformation, cytotoxic, molecular dynamic, anti-inflammatory

## Abstract

Vulgarin, an eudesmanolide sesquiterpene isolated from *Artemisia judaica*, was refluxed with iodine to produce two derivatives (**1** and **2**), which were purified and spectroscopically identified as naproxen methyl ester analogs. The reaction mechanism by which **1** and **2** were formed is explained using a sigmatropic reaction with a 1,3 shift. The scaffold hopping via lactone ring opening enabled the new derivatives of vulgarin (**1** and **2**) to fit well inside the COX-2 active site with ΔG of −7.73 and −7.58 kcal/mol, respectively, which was better than that of naproxen (ΔG of −7.04 kcal/mol). Moreover, molecular dynamic simulations showed that **1** was able to achieve a faster steady-state equilibrium than naproxen. The novel derivative **1** showed promising cytotoxic activities against HepG-2, HCT-116, MCF-7, and A-549 cancer cell lines compared to those of vulgarin and naproxen.

## 1. Introduction

The genus *Artemisia* is one of the most widely distributed of approximately 60 genera in the tribe Anthemideae of the family Astraceae. This genus, with nearly 300 species, is found predominantly in the northern temperate regions of the world [1] and is known to contain many classes of terpenes, including sesquiterpenes. Sesquiterpene lactones are one of the major and attractive bioactive classes due to their complex structures with a wide range of biological activities such as cytotoxic, anti-inflammatory, and antiviral properties [2,3,4,5,6,7,8,9,10,11,12,13]. Because of their structural complexity and biodiversity, they are potential scaffolds for the development of new bioactive compounds [14,15,16]. The eudesmanolide sesquiterpene vulgarin has been reported to occur in numerous *Artemisia* species, including *A. vulgaris* L., after which it was named [17,18]. *A. vulgaris* has been reported to have been used in traditional medicine by the tribal communities in the Western Himalayas to cure rheumatism, stomach problems, hepatic, and sexual disorders [19]. Vulgarin has been assigned other names such as judaicin from *A. judaica* L. [20] and barrelin from *A. barrelieri* Besser. Additionally, it has been isolated from other species including *A. rehan* Chiov. [17], *A. abyssinica* Sch.Bip. ex A.Rich. [21], *A. canariensis* (Besser) Less. [22], and *A. ludoviciana* Nutt. [23]. In addition, vulgarin has been obtained by microbial transformation [24] as well as chemical reduction [21] of peroxyvulgarin [24].

Vulgarin has been reported to be a cytotoxic agent, due to the presence of *α*,*β*-unsaturated ketones [25], an anti-inflammatory agent [26], an oral hypoglycemic agent [27], a cardiotonic agent [28], a powerful central nervous system stimulant, and a potent convulsant poison [20]. The multiple biological activities of vulgarin make it an attractive target for chemo- and biotransformation studies. Previously, we have reported several microbial metabolites of vulgarin [1].

It is known that inflammation acts as a key factor in the development and progression of cancer, since it promotes carcinogenesis, proliferation, and metastasis [29]. Recently, some non-steroidal anti-inflammatory drugs, i.e., NSAIDs, were reported to also have cytotoxic effects [30], with a good correlation between their selectivity and affinity and inhibition of the COX-2 enzyme that results in decreasing prostaglandin E2 synthesis and improvement of their cytotoxic activities [31,32]. Most of the FDA-approved anticancer agents have shown many side effects because of either their ADMET properties or the emergence of resistance. Thus, there is a high-demand need for safe, effective, and potent new anticancer agents [33]. Lead optimization that aims at modifying ligands through chemical and computational approaches to obtain drug-like leads that may have acceptable ADMET properties is a promising technique in the field of drug discovery and development. Among these approaches, scaffold hopping is an important strategy for fulfilling this aim. It can be applied in lead optimization processes to design new chemical entities with better chemical and biological properties [34].

The aim of this work is to develop a new vulgarin-derived scaffold that mimics NSAIDs in their COX-2 affinity and aryl acetic acid scaffold properties with expected cytotoxic activity. The hypothesis is to chemically modify vulgarin to obtain an opened conformation that resembles naproxen with better affinity towards COX-2, and consequently, promising cytotoxic effect.

## 2. Results and Discussion

Computational tools such as docking and in silico screening have recently been used for screening and predicting the cytotoxic activities of novel chemical entities that possess a similar structure to COX-2 inhibitors. This is based on their binding free energy and affinity towards this enzyme [35]. Many of the selective COX-2 inhibitors have been reported to have potential cytotoxic activity against different cell lines [36,37]. The involvement of COX-2 in tumorigenesis and its overexpression in many types of cancer have been the rationale for testing COX-2 inhibitors and/or compounds with high affinity towards COX-2 against cancer cell lines [38]. From this perspective, we computationally investigated two aromatized derivatives of vulgarin via calculating their binding free energy and affinity, and comparing them to a reference COX-2 inhibitor, i.e., naproxen.

The use of molecular iodine to accomplish aromatization of sesquiterpene lactones, with subsequent lactone hydrolysis, has been reported before, for example, santonin [15], ionone [39], and perillaldehyde [40]. In the current report, we show that aromatization and lactone opening of vulgarin using molecular iodine as a catalyst yields two derivatives (**1** and **2**) that are naproxen analogs. This chemical transformation by ring opening is an example of scaffold hopping [34].

The vulgarin used in this project was isolated from *A. abyssinica* and *A. judaica,* as previously described [1]. The isolated vulgarin (132 mg) was refluxed with iodine for 8 h. Then, the reaction was completed and the products were isolated, purified, and identified. The reaction yielded two main derivatives: derivative **1** (10.6 mg, 16.5% yield) and derivative **2** (10.9 mg, 16.7% yield). The reaction was run in a mixture of toluene-MeOH (9:1). This mixture has been reported to be the most efficient one for such a reaction [15]. Moreover, one equivalent of iodine was used, since it has previously been reported to be the optimum quantity [15]. Since iodine also catalyzes esterification, the used methanol yielded the methyl ester derivatives of the two compounds (**1** and **2**) rather than the free acids.

The final products were identified as new naproxen analogs. The structure of derivative **1** was established based on its spectroscopic data. Its molecular formula was determined as C_16_H_18_O_3_ on the basis of the ion peak at *m*/*z* 258.1256 [M]^+^ and ^1^H and ^13^C NMR data (Table 1). The ^13^C NMR spectra (Supplementary Material S2) showed 16 resonances distributed as six singlets, six doublets, and four quartets. When compared to those of vulgarin, on the one hand, it could be observed that compound **1** lacked the two triplets, resonating in vulgarin at ẟ_C_ 23.0 and 34.6, and the three aliphatic doublets, resonating in vulgarin at ẟ_C_ 54.9, 79.9, and 52.7. On the other hand, compound **1** possessed two aromatic doublets, ẟ_C_ 124.7 and 123.2, and three more aromatic singlets, ẟ_C_ 133.6, 122.7, and 138.6, suggesting the aromatization of rings A and B and the opening of lactone ring, ring C. Moreover, compound **1** showed two oxygenated quartet resonances at ẟ_C_ 52.3 and 55.7. Those two carbons correlate, in the HSQC spectra (Supplementary Material S4), to two proton singlets resonating at ẟ_H_ 3.89 and 3.59, respectively, indicating the presence of two methoxy groups. The HMBC spectra (Supplementary Material S5) showed a correlation between the methoxy group resonating at ẟ_H_ 3.59 and assigned to C-16, and C-12 of the carbonyl group resonating at ẟ_C_ 175.3, proving the presence of a methyl ester group. The second methoxy group, on C-14, resonating at ẟ_H_ 3.89 as a singlet, showed a cross peak correlation, in the HMBC spectra, with an aromatic singlet carbon resonating at ẟ_C_ 154.3 which was assigned to C-1. Other HSQC and HMBC data showed the absence of the angular methyl group on C-14 in vulgarin.

The data suggested a concerted sigmatropic 1,3 shift of the methyl group on C-14, from C-10 to the oxygen atom on C-1, during the dienone-phenol rearrangement, with the subsequent aromatization of ring A. The ^1^H NMR spectra of **1** showed five resonances in the aromatic region, one methine quartet in the aliphatic region (ẟ_H_ 3.84, H-11), in addition to three methyl singlets (ẟ_H_ 2.52, 3.59, and 3.89, H-15, 16, and 14, respectively), and a methyl doublet (ẟ_H_ 1.53, H-13).

Likewise, derivative **2** was assigned the molecular formula C_16_H_20_O_3_ as derived from the molecular ion peak at *m*/*z* 260.1404 [M]^+^ and the NMR data (Table 1). The ^13^C NMR spectra (Supplementary Material S9) revealed that compound **2** was similar to **1**, except for the presence of two triplet resonances, ẟ_C_ 24.4 and 21.0, which were assigned as C-8 and C-9, respectively. These assignments were aided by the HMBC spectra (Supplementary Material S12) that showed a correlation between C-8 (ẟ_C_ 24.4) and a proton resonating as a quartet at ẟ_H_ 3.30 which was assigned as H-11. Consequently, H’s-8 were assigned at ẟ_H_ 2.16. The COSY spectrum (Supplementary Material S14) showed coupling contours between H-8 and the other two protons resonating at ẟ_H_ 2.68 and 2.74, which were assigned as H’s-9. The C-9 (ẟ_C_ 21.0) assignment was concluded from the HMBC spectra. Other carbon resonances of **2** were identical, or close to those of **1** (Table 1). 
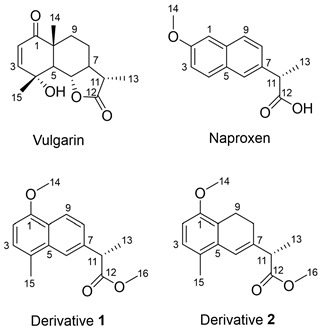

**Table 1:** NMR spectroscopic data (600 MHz, CDCl_3_) of vulgarin and derivatives **1** and **2**.

	Vulgarin		1		2	
#	δ_C_, Type	δ_H_ (*J* in Hz)	δ_C_, Type	δ_H_ (*J* in Hz)	δ_C_, Type	δ_H_ (*J* in Hz)
1	202.1, C ^a^	-	154.3, C	-	154.5, C	-
2	125.9, CH	5.86, d (10.4)	103.6, CH	6.61, d (7.8)	109.6, CH	6.58, d (8.4)
3	152.2, CH	6.58, d (10.4)	126.8, CH	7.12, d (7.8)	128.3, CH	6.86, d (8.4)
4	70.4, C	-	126.1, C	-	125.8, C	-
5	54.9, CH	2.40, d (11.5)	133.6, C	-	133.4, C	-
6	79.9, CH	4.15, dd (10.9, 10.9)	122.7, CH	7.72, d (1.2)	121.3, CH	6.43, dd (2.4, 1.2)
7	52.7, CH	1.67, dddd (12.6, 12.6, 12.6, 3.5)	138.6, C	-	140.1, C	-
8	23.0, CH_2_	1.96, m 1.46, dddd (12.9, 12.9, 12.9, 3.2)	124.7, CH	7.36, dd (8.4, 1.8)	24.4, CH_2_	2.16, m 2.16, m
9	34.6, CH_2_	1.99, m 1.56, ddd (13.6, 13.6, 3.5)	123.2, CH	8.17, d (8.4)	21.0, CH_2_	2.74, m 2.68, m
10	46.6, C	-	125.0 C	-	123.0, C	-
11	40.9, CH	2.34, dq (13.7, 6.9)	46.1, CH	3.84, q (7.2)	47.1, CH	3.30, q (7.8)
12	178.7, C		175.3, C	-	174.9, C	-
13	12.8, CH_3_	1.22, d (6.9)	18.9 ^b^, CH_3_	1.53, d (6.6)	15.9, CH_3_	1.31, d (7.2)
14	20.1, CH_3_	1.19, s	55.7, CH_3_	3.89, s	55.8, CH_3_	3.72, s
15	24.1, CH_3_	1.53, s	19.0 ^b^, CH_3_	2.52, s	18.7, CH_3_	2.20, s
16	-	-	52.3, CH_3_	3.59, s	52.1, CH_3_	3.62, s

^a^ Carbon multiplicities were determined by DEPT 135°; ^b^ assignments bearing the same superscript within the same column are interchangeable.

**Scheme 1:**
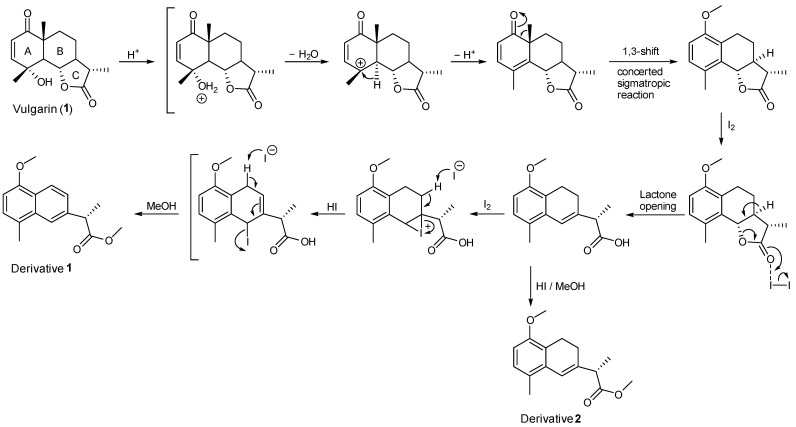
The proposed mechanism involved in the formation of derivatives **1** and **2**. A concerted sigmatropic reaction involving a 1,3 shift is proposed.

The derivatives were produced via an iodine-catalyzed aromatization mechanism [41], where molecular iodine (I_2_) decomposed to generate the acidic conditions (HI) needed for dienone-phenol rearrangement to occur, with the successive aromatization. The used protic solvent, MeOH, facilitated the formation of HI acid that protonated the oxygen of the carbonyl group, resulting in the four-centered concerted reaction, 1,3-methyl sigmatropic shift, that ended up with the formation a double bond between C-1 and C-10, as seen in the reaction scheme (Scheme 1).

Additionally, the acid-catalyzed dehydration of the hydroxy group on C-4 led to the aromatization of ring A. Simultaneously, the iodine-catalyzed lactone opening produced a double bond between C-6 and C-7 in ring B, followed by esterification to afford the methyl ester as derivative **2** (Scheme 1). Furthermore, the intermediate iodirane may undergo several iodination and dehydroiodination steps that lead to a complete aromatization, followed by esterification to produce derivative **1** (Scheme 1).

**Figure 1:**
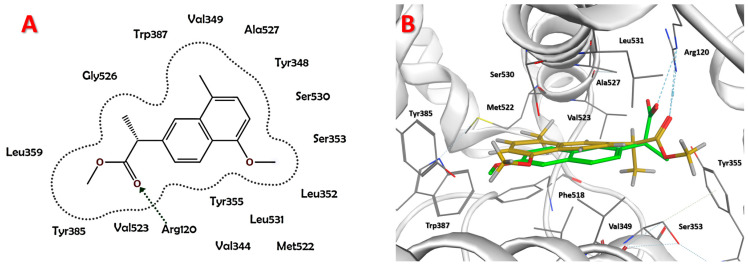
(**A**) 2D placement of derivative **1** inside the active site of COX-2; (**B**) similar orientation of naproxen in a 3D view (green) and derivative **1** (yellow), they are superimposed by their docking poses.

Derivatives **1** and **2**, vulgarin, and naproxen were subjected to a docking process in the main active site at which naproxen was crystalized and the free energy of binding was assessed by London ΔG, affinity ΔG, and GBVI/WSA ΔG scores (Table 2). The 2D pose of derivative **1** showed the aryl acetic acid scaffold that mimics naproxen, as the element that enabled derivative **1** to fit well in the active site of COX-2 via forming a hydrogen bond with Arg120 (Figure 1A). Naproxen and derivative **1** both confirmed the same orientation mode of the carboxylate towards interacting with Arg120 residue of the COX-2 active site (Figure 1B).

**Figure 2:**
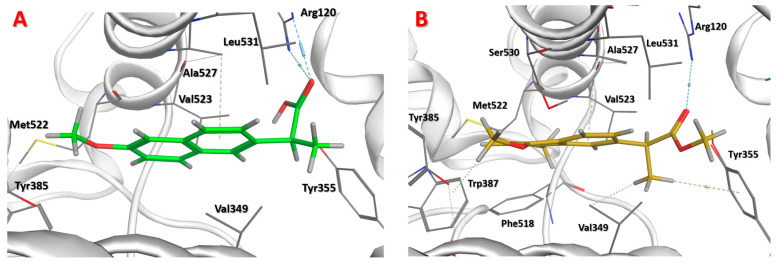
(**A**) Best docking pose of naproxen; (**B**) best docking pose of derivative **1**.

Naproxen was subjected to a redocking process to evaluate its binding mode. It showed two hydrogen bonds with Arg120 and Van der Waals interactions with Ala527 (Figure 2A). Docking of derivative **1** showed more pharmacodynamic interactions than naproxen. The oxygen of the carboxylate group formed a hydrogen bond with Arg120, the 16-methoxy group formed hydrophobic interactions with Trp387, and the 13α-methyl group showed hydrophobic interactions with both Tyr355 and Val349 (Figure 2B). This increase in the pharmacodynamic interactions can predict higher binding affinity, stability, and better biological activity than naproxen itself.

**Figure 3:**
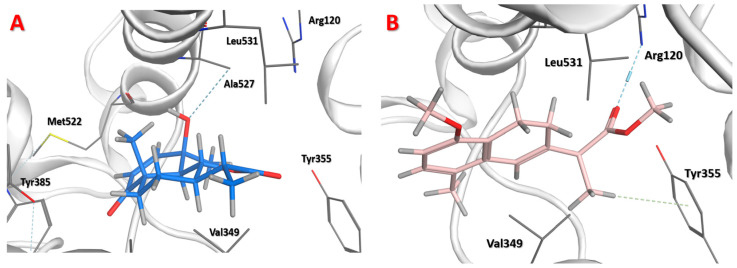
(**A**) Best docking pose of vulgarin shows the interaction with Ala527; (**B**) docking pose of derivative **2**.

The docking of vulgarin showed only one hydrogen bond with Ala527 (Figure 3) due to its rigid structure that does not allow much flexibility, in particular, for the carbonyl of the lactone to interact with Arg120 (the key interaction residue). Derivative **2** showed a hydrogen bond with Arg120 and Van der Waals interactions between the 13α-methyl group and Tyr355 (Figure 3B). Derivative **2** lacked the planarity of naphthalene due to the absence of C=C between C-8 and C-9, which resulted in a different conformation than that of derivative **1**. In addition, derivative **2** was not able to show a superimposition with naproxen similar to that of derivative **1**.

**Figure 4:**
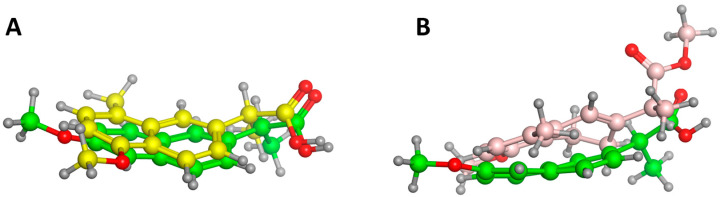
(**A**) Superimposition of naproxen (green) with derivative **1** (yellow); (**B**) superimposition of naproxen (green) with derivative **2** (pink).

Derivative **1** with naphthyl acetic acid scaffold showed a better superimposition with naproxen with the same scaffold (Figure 4A). However, the absence of one double bond in derivative **2** caused a conformational change that resulted in more deviation when superimposed with naproxen (Figure 4B).

The binding site of COX-2 is surrounded by many residues, namely, Arg120, Val523, Ala523, Tyr385, Leu359, Gly526, Trp387, Val349, Ala527, Try348, Ser530, Ser353, Leu352, Leu531, Tyr355, Met522, and Val344 (Figure 1A), however, the only reported interaction of COX-2 inhibitors is the one between their carboxylate groups and Arg120 [42].

The pharmacodynamic approach employed in this study is a widely used approach among the optimization methods, in which the aim is to increase the ligand–enzyme interactions. Derivative **1** achieved this aim by increasing the site interaction, as explained previously, compared to naproxen. Moreover, the computed affinity ΔG score of **1** was found to be close to that of naproxen (1.08 Å) (Table 2), whereas London ΔG (−7.73) and GBVI/WSA ΔG (−20.01) of derivative **1** were more than that of naproxen.

According to the docking results, derivative **1** was the top ranked in all scores, with more pharmacodynamic interactions. This encouraged us to perform a molecular dynamics simulation study before testing its in vitro cytotoxic activity, to evaluate the binding stability of this derivative and to compare it to that of naproxen since this can be used as a validation for the docking results.

**Figure 5:**
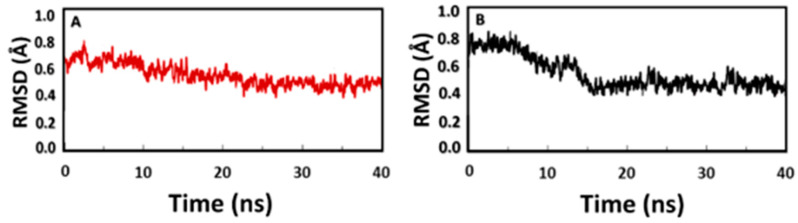
Molecular dynamics results of: (**A**) naproxen; (**B**) derivative **1**.

The main aim of the molecular dynamics study was to validate the binding stability and strength of derivative **1** compared to those of naproxen. The molecular dynamics simulation was conducted over a 40 ns period, and, on the one hand, showed that naproxen exhibited standard oscillations started at 0.0 ns with an RMSD value of 0.6 Å from its best docking pose, and then the RMSD value started to decrease gradually until the equilibrium was achieved at 20 ns with an RMSD value of 0.5 Å, when the steady state was observed (Figure 5A). On the other hand, derivative **1** started at an RMSD value of 0.8 Å and decreased until 0.5 Å, during the first 10 ns. Then, a slight increase was observed (RMSD = 0.6 Å), followed by a decline at 15 ns, where the steady state was reached with an RMSD value of 0.4 Å (Figure 5B).

The molecular dynamics results confirmed that derivative **1** reached its steady-state equilibrium after a shorter time (15 ns) than naproxen which reached its equilibrium after 20 ns. In addition, at the point of equilibrium, derivative **1** conformation had an RMSD = 0.4 Å, while naproxen showed an RMSD = 0.5 Å. For these reasons, derivative **1** is considered to be more stable than naproxen.

Derivatives **1** and **2**, as well as vulgarin were evaluated for their cytotoxic activities against hepatocellular (HepG-2), colon (HCT-116), breast (MCF-7), and lung (A-549) carcinoma cell lines. Derivatives **1** and **2** were shown to be more active than vulgarin against all tested cell lines (Table 3). Derivative **1** showed the highest activity against the HepG-2 cell line with CC_50_ values of 151 ± 11 µM. Likewise, it was the most potent derivative against all tested cell lines (Table 3). The CC_50_ values obtained for derivative **1** were shown to be lower, by approximately eight-fold, than those reported for naproxen against the same cell lines; >1.5 mM vs. HCT-116 [43], 2.3 mM vs. MCF-7 [44], and >5 mM [45] and >10 mM [46] vs. A-549.

The high cytotoxic activities of **1** compared to those of the other compounds (derivative **2**, vulgarin, and naproxen) were in accordance with the computed high docking affinity with a low RMSD and high molecular dynamics stability for this derivative. Moreover, derivatives **1** and **2** as well as vulgarin were shown to be more active against all tested cancer cells compared to naproxen, results that were consistent with the computed docking affinity and molecular dynamics stability for those compounds.

The anti-inflammatory activity of the tested compounds was predicted using the InflamNat online platform which contains a database of 1351 compounds with reported anti-inflammatory activity. The InflamNat platform also contains all data about cell-based anti-inflammatory bioassays, assays in inflammatory cell models, and information about the production of inflammatory factors and cell cytokines. According to the dataset model implemented in InflamNat, a compound is considered to be active if its predicted IC_50_/EC_50_ values are <50 µM. An inactive compound has IC_50_/EC_50_ values >50 µM. Therefore, InflamNat is a powerful tool for the prediction of anti-inflammatory activity. Here, we used the SMILES of all tested compounds, and the results were very promising compared to those of naproxen (Table 4). Derivative **1** showed an IC_50_ value of 0.34 µM which was very close to that of naproxen (0.33 µM). All compounds showed predicted values <50 µM.

## 3. Materials and Methods

### 3.1. General Experimental Procedure

The IR spectra were recorded as a chloroform film using an FT/IR-4100 type A spectrophotometer. The ^1^H and ^13^C NMR spectra (Supplementary Materials) were obtained on a Bruker Avance II-600 spectrometer operating at 600 and 150 MHz, respectively. The ^1^H and ^13^C NMR spectra were both recorded in CDCl_3_, and the chemical shift values were expressed in *d* (ppm) relative to the internal standard TMS. For the ^13^C NMR spectra, spectral editing was determined by using DEPT. The 2D NMR data (Supplementary Materials) were obtained using the standard pulse sequence of the Bruker Avance II-600 for COSY, HSQC, and HMBC. The HREIMS analysis was carried out on a high-resolution GC/MS-DFS (Double Focusing Sector). Column chromatography was carried out on silica gel 60 (230–400 mesh ASTM, Merck, Darmstadt, Germany). The TLC analysis was carried out on silica gel 60 F254 (Merck, Darmstadt, Germany) plates. Compounds were detected by using UV and *p*-anisaldehyde/H_2_SO_4_ spraying reagent followed by heating at 105 °C for 1–2 min.

### 3.2. Plant Material

The vulgarin used in this project was isolated, as reported before [21], from *A. abyssinica*, and *A. judaica* which were collected from the Tabouk area, Saudi Arabia, in December 2002. The plants were identified, and voucher specimens were deposited at the herbarium of the Medicinal Aromatic and Poisonous Plants Research Center, College of Pharmacy, King Saud University, Riyadh, Saudi Arabia.

### 3.3. Synthesis of Derivatives ***1*** and ***2***

A previously reported method [41] after slight modification was applied. Briefly, vulgarin (0.132 g, 0.5 mmol) and iodine (0.379 g, 1.5 mmol) were mixed in 5 mL of toluene-methanol (9:1). The mixture was refluxed for 8 h and monitored by TLC. Ethyl acetate and water containing sodium thiosulfate (0.047 g, 3 mmol in 100 mL H_2_O) were used to terminate the reaction. The organic layer was collected, washed with water, passed over anhydrous sodium sulfate, and finally evaporated in vacuo to give 103.2 mg of a syrupy residue.

### 3.4. Purification of Derivatives ***1*** and ***2***

The obtained residue was chromatographed over a silica gel column (12 g, 17 × 1.5 cm) using hexane/chloroform (1:1) as the eluting solvent. Fractions (4 mL each) were collected. Similar fractions, based on their TLC appearance, were pooled together to give 7 fractions (A–G). Fraction B (42 mg) was further purified over a silica gel column (6 g, 13 × 1.5 cm), and eluted initially with 40% *n*-hexane/toluene, followed by a 100% toluene. Similar fractions were pooled together to afford 10.6 mg of **1** and 10.9 mg of **2**.

Derivative **1**: colorless gummy residue; IR (neat) n_max_ 2950, 1734, and 1204 cm^−1^; ^1^H NMR (CDCl_3_, 600 MHz) see Table 1; ^13^C NMR (CDCl_3_, 150 MHz) see Table 1; ESIMS *m*/*z* 258.28 [M]+ (98); HRESIMS *m*/*z* 258.1256 (calculated for C_16_H_18_O_3_, 258.1250).

Derivative **2**: colorless gummy residue; IR (neat) n_max_ 2949, 1733, and 1204 cm^−1^; ^1^H NMR (CDCl_3_, 600 MHz) see Table 1; ^13^C NMR (CDCl_3_, 150 MHz) see Table 1; ESIMS *m*/*z* 260.28 [M]+ [44]; HRESIMS *m*/*z* 260.1404 (calculated for C_16_H_20_O_3_, 260.1407).

### 3.5. Molecular Docking Studies

The molecular docking studies were conducted using the Molecular Operating Environment package license (Molecular Operating Environment 2022.02, Chemical Computing Group Inc., Montreal, QC, Canada). Triangle matcher was used as a placement method. Free energy of binding was evaluated using London DG, affinity DG, and GBVI/WSA DG scores. The crystal structure of COX-2 in complex with naproxen (pdb code = 3NT1) [42] was downloaded from a protein data bank (https://www.rcsb.org, accessed on 30 November 2022). This protein was resolved by X-ray crystallography method with resolution = 1.73 Å and R value = 0.186.

### 3.6. Molecular Dynamics Simulations

The docking of both naproxen and derivative **1** revealed a stable pose that was kept in the active site. The protein geometries, electron density, and temperature-related factors were prepared. All hydrogens were added, and energy minimization was calculated. Any foreign solvent molecules in the system were deleted. Then, salt atoms were added to the system to surround the biomolecular protein–ligand complex in a spherical shape. Sodium chloride was added to a concentration of 0.1 M. The cell dimensions were 100.309 × 89.2061 × 81.4899 Å, and its shape was 90 × 90 × 90 Å. The total number of solvent molecules within the system was 21269; 1.023 g/cm^3^. Assisted Model Building with Energy Refinement 10: Extended Hückel Theory (AMBER 10: EHT) was selected as a force field with an R-Field of 1:80/VdW. The heat was adjusted in order to increase the temperature of the system from 0 to 300 °K, which was followed by equilibration and production for 300 ps. Then, cooling was initiated until 0 °K was reached. The molecular dynamics protocol used the Nose-Poincare-Andersen algorithm to solve the equation of motion. The simulation was conducted over a 40 ns time period (40,000 ps) using Molecular Operating Environment 2022.02.

### 3.7. Cytotoxicity Assay

This assay was conducted at the Regional Center for Mycology and Biotechnology, Al-Azhar University, Cairo, Egypt, where cancer cells were grown on RPMI-1640 medium supplemented with 10% inactivated fetal calf serum and 50 µg/mL of gentamycin. The cells were maintained at 37 °C in a humidified atmosphere with 5% CO_2_ until they were used.

Vulgarin and its derivatives **1** and **2** were evaluated for their cytotoxic activities against liver (HepG-2), colon (HCT-116), breast (MCF-7), and lung (A-549) carcinoma cell lines using “cell viability assays” [47]. Cancer cells were suspended in media at a concentration of 5 × 10^4^ cell/well in 96-well plates, then incubated for 24 h before treatment with the test compounds. Test compounds were added to the wells (triplicates) to achieve twelve concentrations for each compound. Vehicle controls with media or 0.5% DMSO were used. After 24 h of incubation, the numbers of viable cells were determined by MTT assay. Briefly, the media were replaced with 100 µL of fresh RPMI-1640 medium, and 10 µL of 12 mM MTT stock solution (5 mg of MTT in 1 mL of PBS) was added to the untreated control wells. Then, the 96-well plates were incubated at 37 °C and 5% CO_2_ for 4 h; 85 µL aliquots of the media was removed from the wells; 50 µL of DMSO was added to each well and mixed thoroughly, and incubated at 37 °C for 10 min. Then, the optical density was measured at 590 nm with the microplate reader (SunRise, TECAN Inc., Morrisville, NC, USA). The viability percentage was calculated and the CC_50_ values (µM) were estimated from graphic plots of the dose-response curve for each concentration (Table 3) using the GraphPad Prism software (version 9.5.1.733, San Diego, CA, USA) [48].

### 3.8. Web-Based Prediction of the Anti-Inflammatory Activity

All compounds (vulgarin, derivatives **1** and **2**, and naproxen) were drawn using MOE 2022.02, and their SMILES were copied and used in the InflamNat web [49]. The results obtained were in IC_50_ (µM).

## 4. Conclusions

In this study, two novel vulgarin derivatives were synthesized through a four-centered concerted reaction mechanism, where a 1,3-methyl sigmatropic shift was involved. These two derivatives were found to be naproxen methyl ester analogs. These derivatives, particularly derivative **1**, were shown to possess an arylacetic acid scaffold that mimics NSAIDs in their COX-2 affinity with possible biological activities. Derivative **1** showed better London ΔG, GBVI/WSA ΔG, and affinity ΔG scores when compared to naproxen. In addition, it exhibited a higher cytotoxic activity against HepG-2, HCT-116, MCF-7, and A-549 cell lines than those reported for naproxen. The scaffold-hopping ring opening of vulgarin produced two novel derivatives yet to be investigated for more biological activities.

## Figures and Tables

**Table 2:** Computed docking results of the investigated compounds compared to naproxen using pdb = 3NT1.

Compounds	London ΔG (kcal/mol)	GBVI/WSA ΔG	Affinity ΔG
Vulgarin	−6.74	−18.09	−7.87
Derivative **1**	−7.73	−20.01	−10.13
Derivative **2**	−7.58	−19.11	−8.20
Naproxen	−7.04	−19.45	−9.82

**Table 3:** CC_50_ values (mean ± SD) of vulgarin and derivatives **1** and **2**.

Cell Lines	CC_50_ (µM)		
	Vulgarin	1	2
HepG-2	770 ± 23	151 ± 11	540 ± 23
HCT-116	723 ± 24	207 ± 11	310 ± 13
MCF-7	1061 ± 36	304 ± 13	925 ± 28
A-549	835 ± 27	264 ± 15	472 ± 16

**Table 4:** InflamNat predicted IC_50_ values for the anti-inflammatory activity.

Compounds	Predicted IC_50_ (µM)
Vulgarin	0.65
Derivative **1**	0.34
Derivative **2**	0.45
Naproxen	0.33

## Data Availability

Data is contained within the article or Supplementary Materials.

## Supplementary Materials

The following supporting information can be downloaded at: https://www.mdpi.com/article/10.3390/molecules28083421/s1, ^1^H, ^13^C, DEPT 45°, DEPT 90°, DEPT 135°, HSQC, HMBC, and COSY spectra of vulgarin and derivatives **1** and **2**.

## Sample Availability

Samples of the compounds are available from the authors.
