# Supplementary material for: Novel Vulgarin Derivatives: Chemical Transformation, In Silico and In Vitro Studies
Source: Molecules. 2023 Apr 13;28(8):3421. doi: 10.3390/molecules28083421 (PMC10143240; doi:10.3390/molecules28083421)
Supplement: Supplementary file 1 [file molecules-28-03421-s001.zip › molecules-2317356-supplementary.pdf]

Novel Vulgarin Derivatives: Chemical Transformation, *In Silico* and *In Vitro* Studies

Hanan G. Sary [1, 2], Mohammed A. Khedr [1] and Khaled Y. Orabi [1*]

[1] Department of Pharmaceutical Chemistry, College of Pharmacy, Kuwait University, 13110 Safat, Kuwait; hanan.sary@ku.edu.kw, mohammed.khedr@ku.edu.kw, ky.orabi@ku.edu.kw

[2] Department of Pharmacognosy, Faculty of Pharmacy, Ain-Shams University, Cairo, Egypt; hanangaber@pharma.asu.edu.eg

* Correspondence: ky.orabi@ku.edu.kw; Tel.: ((965) 2 463-6158)

CONTENTS

S1 ^{1}H NMR (600 MHz, $CDCl_3$) spectrum of derivative **1**

S2 ^{13}C NMR (600 MHz, $CDCl_3$) spectrum of derivative **1**

S3 DEPT-135 (600 MHz, $CDCl_3$) spectrum of derivative **1**

S4 HSQC (600 MHz, $CDCl_3$) spectrum of derivative **1**

S5 HMBC (600 MHz, $CDCl_3$) spectrum of derivative **1**

S6 HMBC (600 MHz, $CDCl_3$) spectrum of derivative **1**

S7 COSY (600 MHz, $CDCl_3$) spectrum of derivative **1**

S8 ^{1}H NMR (600 MHz, $CDCl_3$) spectrum of derivative **2**

S9 ^{13}C NMR (600 MHz, $CDCl_3$) spectrum of derivative **2**

S10 DEPT-135 (600 MHz, $CDCl_3$) spectrum of derivative **2**

S11 HSQC (600 MHz, $CDCl_3$) spectrum of derivative **2**

S12 HMBC (600 MHz, $CDCl_3$) spectrum of derivative **2**

S13 HMBC (600 MHz, $CDCl_3$) spectrum of derivative **2**

S14 COSY (600 MHz, $CDCl_3$) spectrum of derivative **2**

S1. ^{1}H NMR (600 MHz, $CDCl_3$) spectrum of derivative **1**

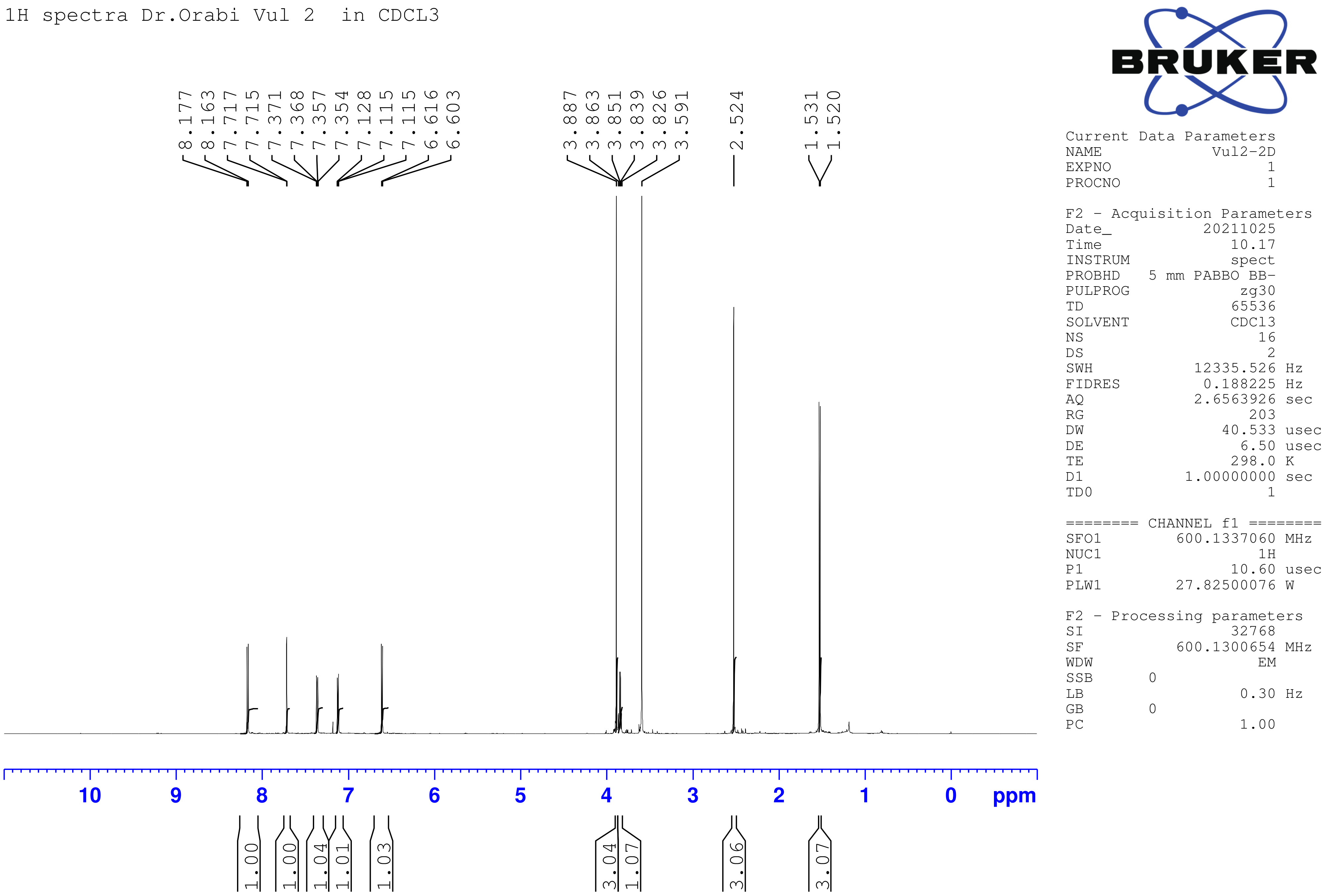

S2. ^{13}C NMR (600 MHz, $CDCl_3$) spectrum of derivative **1**

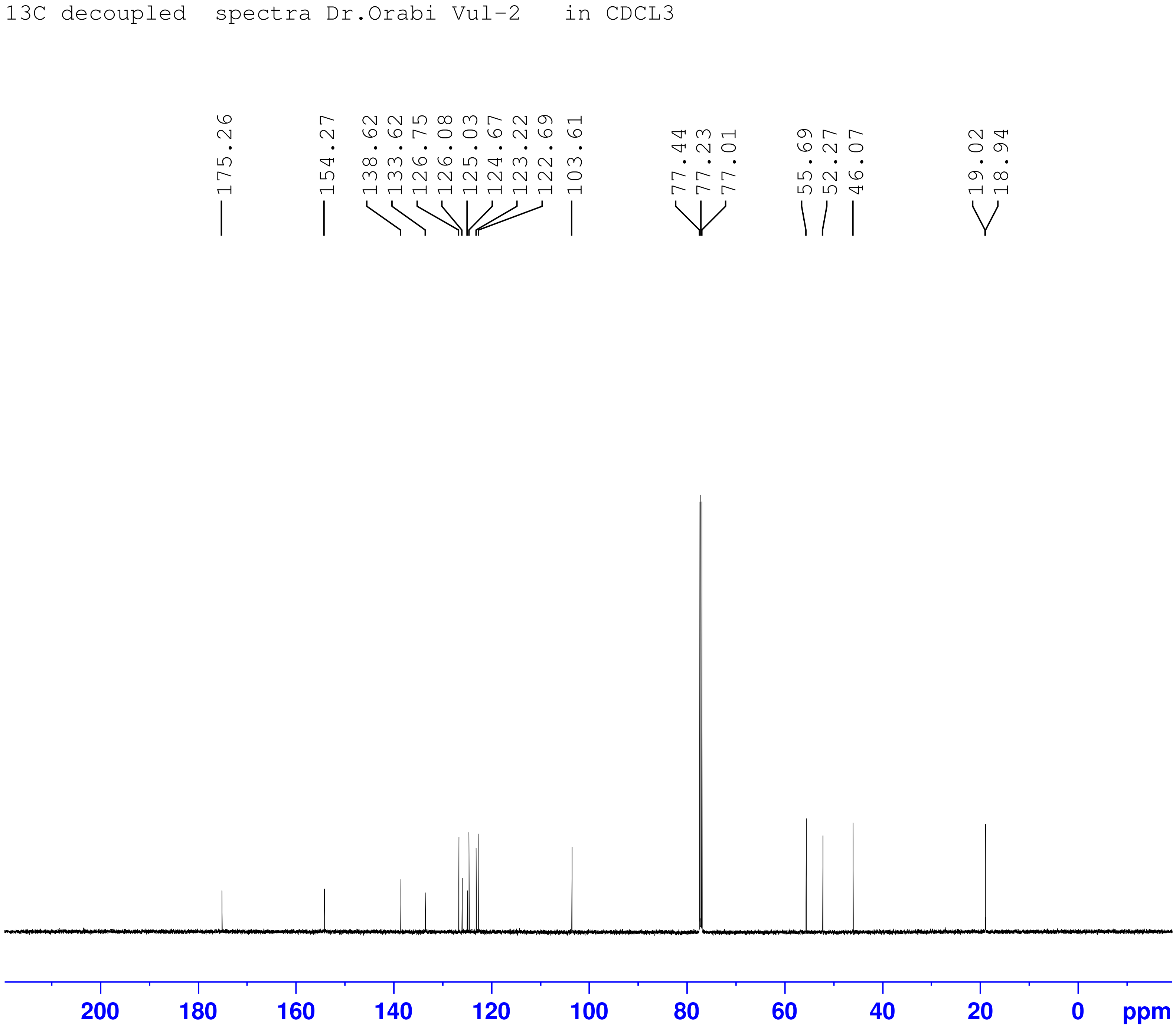

Current Data Parameters	
NAME	Vul2-2D
EXPNO	2
PROCNO	1
F2 - Acquisition Parameters	
Date_	20211025
Time	10.30
INSTRUM	spect
PROBHD	5 mm PABBO BB-
PULPROG	zgpg30
TD	65536
SOLVENT	CDCl3
NS	520
DS	4
SWH	36057.691 Hz
FIDRES	0.550197 Hz
AQ	0.9087659 sec
RG	203
DW	13.867 usec
DE	50.00 usec
TE	298.0 K
D1	2.00000000 sec
D11	0.03000000 sec
TD0	1
======== CHANNEL f1 ========	
SFO1	150.9178979 MHz
NUC1	13C
P1	8.80 usec
PLW1	78.13500214 W
======== CHANNEL f2 ========	
SFO2	600.1324005 MHz
NUC2	1H
CPDPRG[2	waltz65
PCPD2	70.00 usec
PLW2	27.82500076 W
PLW12	0.63804001 W
PLW13	0.32093000 W
F2 - Processing parameters	
SI	32768
SF	150.9027796 MHz
WDW	EM
SSB	0
LB	1.00 Hz
GB	0
PC	1.40

S3. DEPT-135 (600 MHz, $CDCl_3$) spectrum of derivative **1**

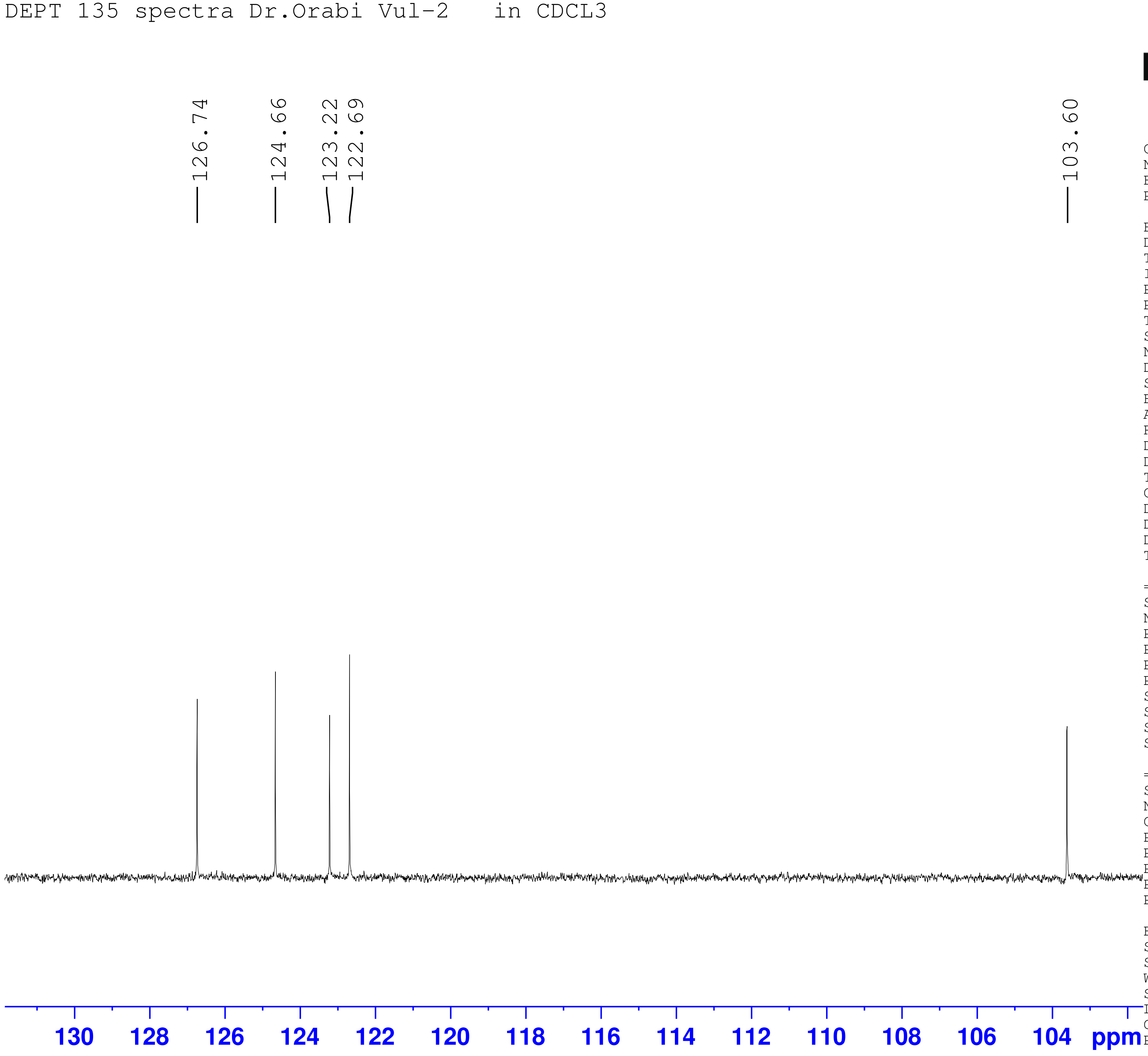


```
Current Data Parameters
NAME              Vul2-2D
EXPNO                   5
PROCNO                  1

F2 - Acquisition Parameters
Date_            20211025
Time                11.45
INSTRUM             spect
PROBHD   5 mm PABBO BB-
PULPROG         deptsp135
TD                  65536
SOLVENT             CDCl3
NS                    169
DS                      4
SWH             36057.691 Hz
FIDRES           0.550197 Hz
AQ              0.9087659 sec
RG                    203
DW                 13.867 usec
DE                  50.00 usec
TE                  298.0 K
CNST2         145.0000000
D1             2.00000000 sec
D2             0.00344828 sec
D12            0.00002000 sec
TD0                     1

======== CHANNEL f1 ========
SFO1          150.9178979 MHz
NUC1                  13C
P1                   8.80 usec
P13               2000.00 usec
PLW0         0 W
PLW1          78.13500214 W
SPNAM[5]      Crp60comp.4
SPOAL5              0.500
SPOFFS5      0 Hz
SPW5           9.24489975 W

======== CHANNEL f2 ========
SFO2          600.1324005 MHz
NUC2                   1H
CPDPRG[2          waltz65
P3                  10.60 usec
P4                  21.20 usec
PCPD2               70.00 usec
PLW2          27.82500076 W
PLW12          0.63804001 W

F2 - Processing parameters
SI                  32768
SF            150.9027803 MHz
WDW                    EM
SSB          0
LB                   1.00 Hz
GB           0
PC                   1.40
```

S4. HSQC (600 MHz, $CDCl_3$) spectrum of derivative **1**

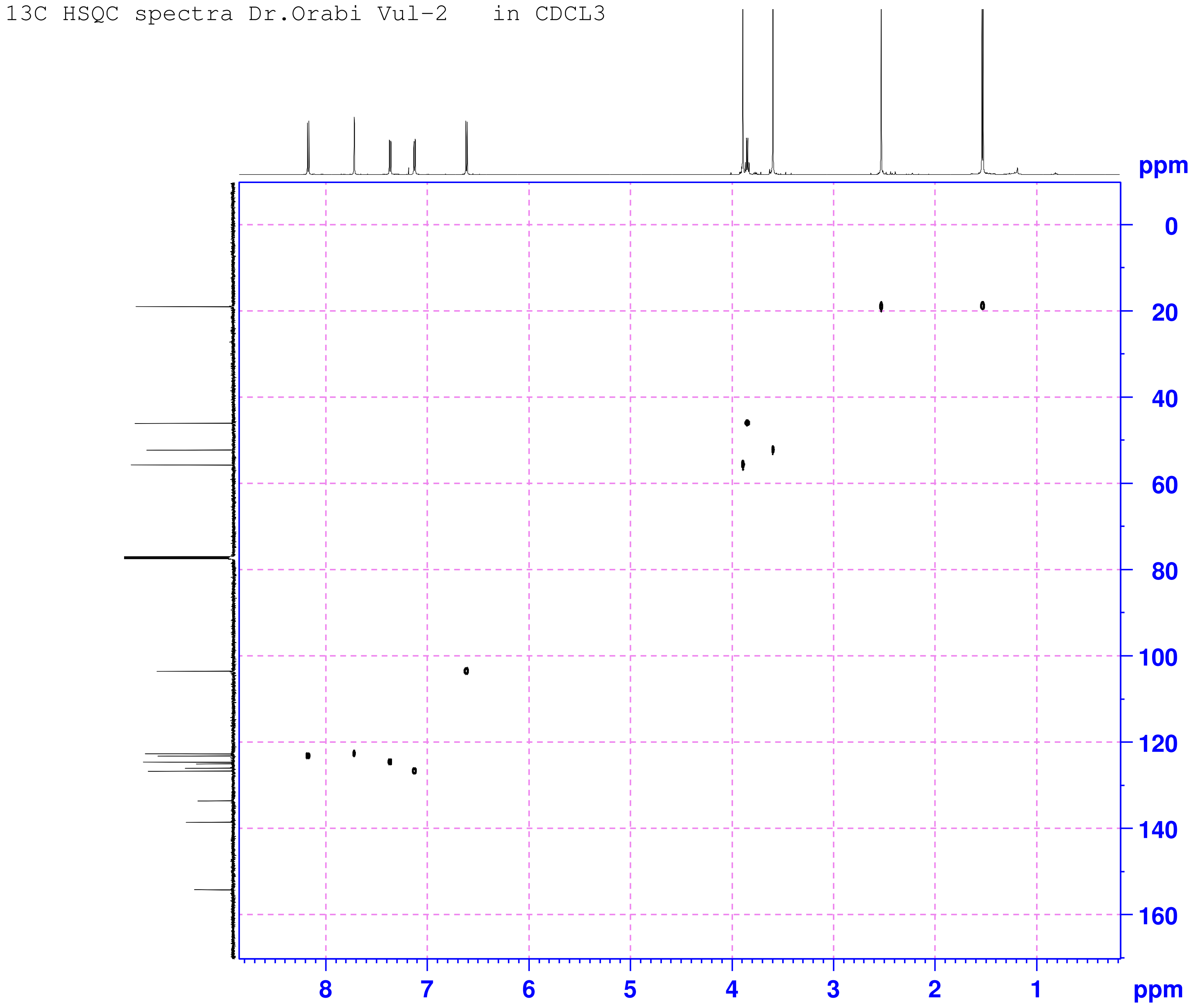


```
Current Data Parameters
NAME              Vul2-2D
EXPNO                   7
PROCNO                  1

F2 - Acquisition Parameters
Date_            20211025
Time                12.11
INSTRUM             spect
PROBHD   5 mm PABBO BB-
PULPROG    hsqcetgpsp.2
TD                   2048
SOLVENT             CDCl3
NS                      4
DS                     16
SWH              5208.333 Hz
FIDRES           2.543132 Hz
AQ              0.1966080 sec
RG                    203
DW                 96.000 usec
DE                   6.50 usec
TE                  298.1 K
CNST2         145.0000000
D0             0.00000300 sec
D1             1.90988803 sec
D4             0.00172414 sec
D11            0.03000000 sec
D16            0.00020000 sec
IN0            0.00001840 sec
ZGOPTNS

======== CHANNEL f1 ========
SFO1          600.1327722 MHz
NUC1                   1H
P1                  10.60 usec
P2                  21.20 usec
P28               1000.00 usec
PLW1          27.82500076 W

======== CHANNEL f2 ========
SFO2          150.9148803 MHz
NUC2                  13C
CPDPRG[2            garp4
P3                   8.80 usec
P14                500.00 usec
P24               2000.00 usec
PCPD2               60.00 usec
PLW0       0 W
PLW2          78.13500214 W
PLW12          1.68079996 W
SPNAM[3] Crp60,0.5,20.1
SPOAL3              0.500
SPOFFS3    0 Hz
SPW3           9.24489975 W
SPNAM[7]     Crp60comp.4
SPOAL7              0.500
SPOFFS7    0 Hz
SPW7           9.24489975 W

====== GRADIENT CHANNEL =====
GPNAM[1]         SINE.100
GPNAM[2]         SINE.100
GPZ1                80.00 %
GPZ2                20.10 %
P16               1000.00 usec

F1 - Acquisition parameters
TD                    187
SFO1             150.9149 MHz
FIDRES         145.315048 Hz
SW                180.061 ppm
FnMODE       Echo-Antiecho

F2 - Processing parameters
SI                   2048
SF            600.1300640 MHz
WDW                 QSINE
SSB                     2
LB         0 Hz
GB         0
PC                   1.40

F1 - Processing parameters
SI                   1024
MC2          echo-antiecho
SF            150.9027671 MHz
WDW                 QSINE
SSB                     2
LB         0 Hz
GB         0
```

S5. HMBC (600 MHz, $CDCl_3$) spectrum of derivative **1**

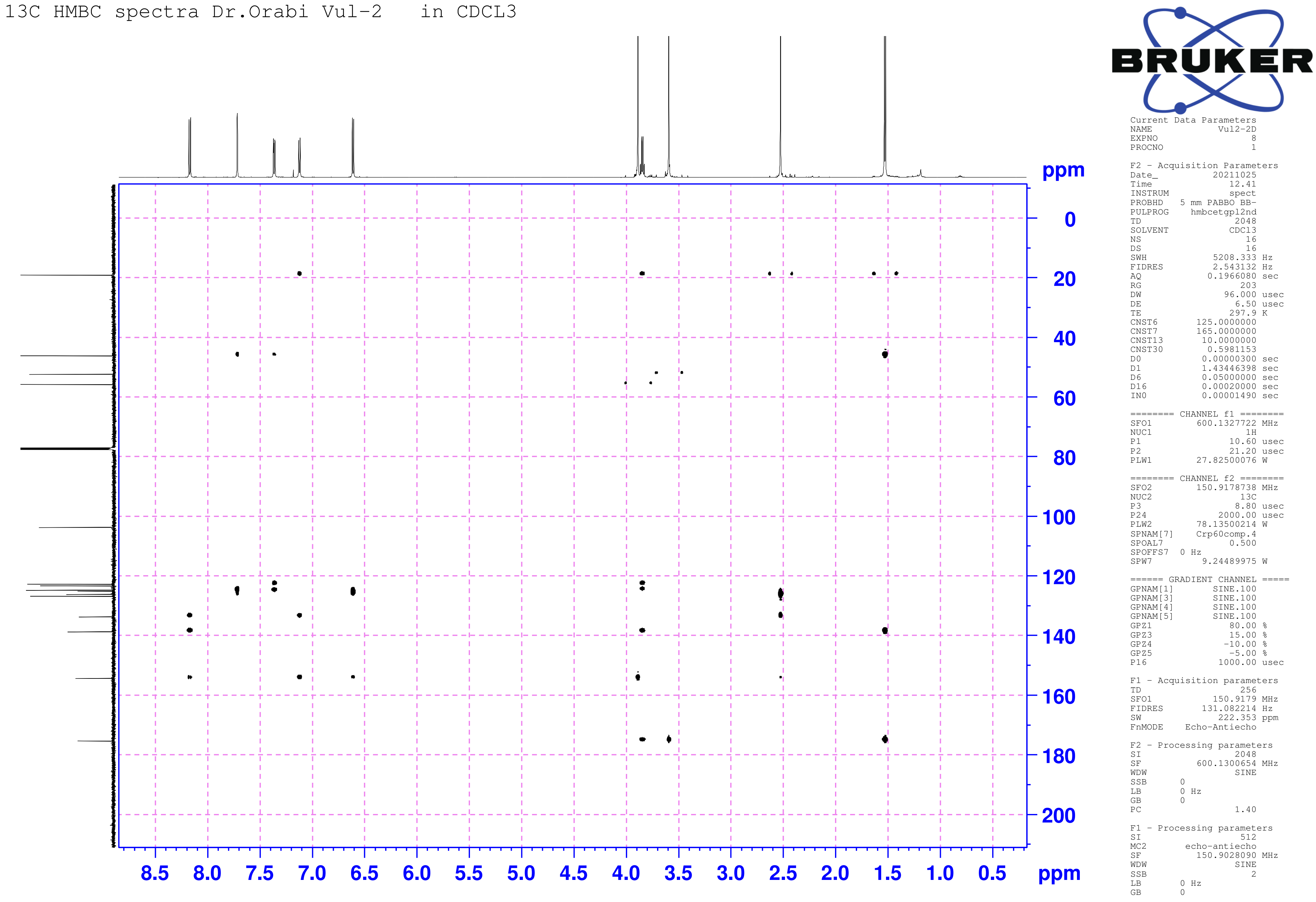

S6. HMBC (600 MHz, $CDCl_3$) spectrum of derivative **1**

13C HMBC spectra Dr.Orabi Vul-2   in CDCL3

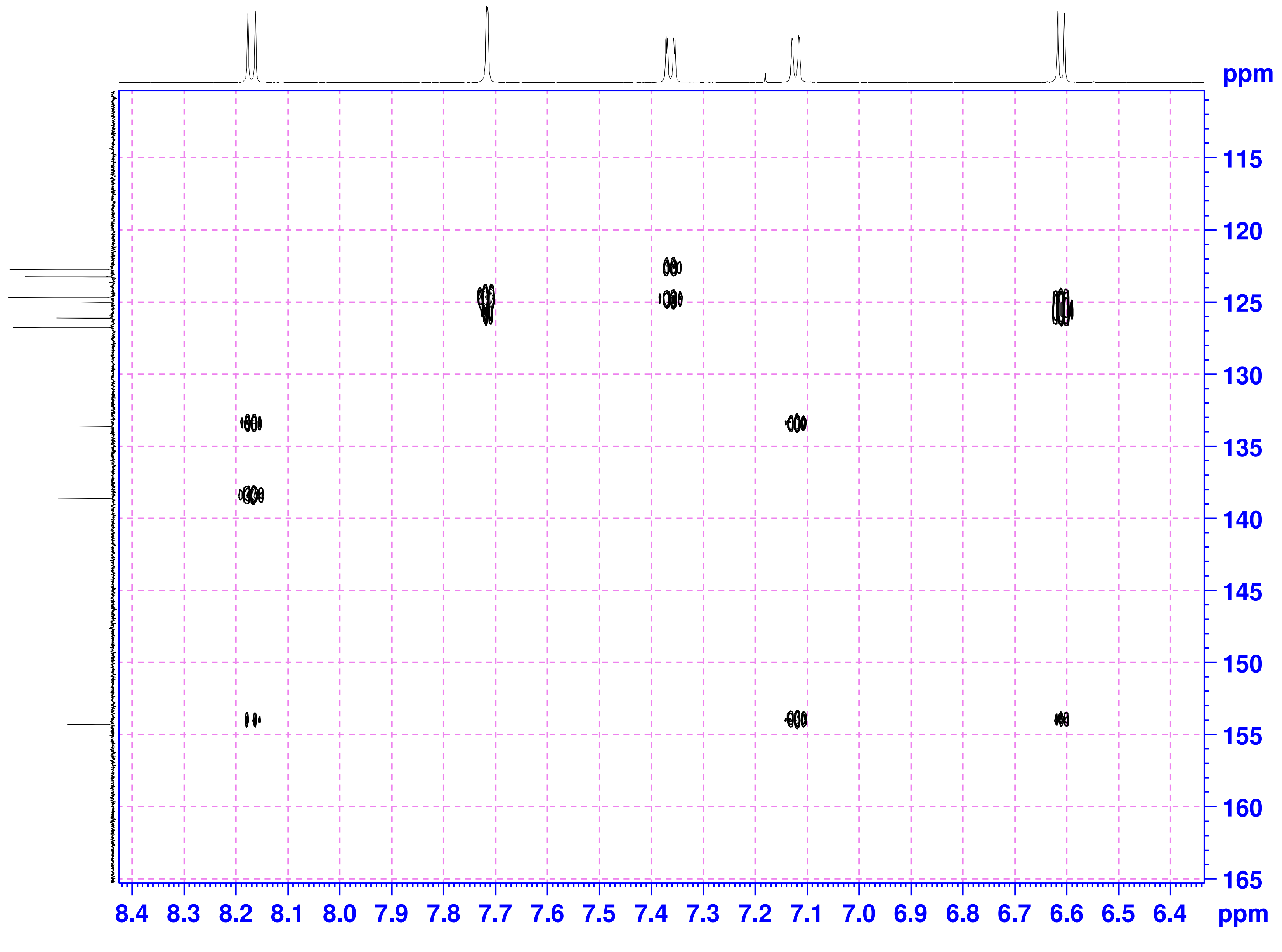


```
Current Data Parameters
NAME              Vul2-2D
EXPNO                   8
PROCNO                  1

F2 - Acquisition Parameters
Date_            20211025
Time                12.41
INSTRUM             spect
PROBHD   5 mm PABBO BB-
PULPROG    hmbcetgpl2nd
TD                   2048
SOLVENT             CDCl3
NS                     16
DS                     16
SWH              5208.333 Hz
FIDRES           2.543132 Hz
AQ              0.1966080 sec
RG                    203
DW                 96.000 usec
DE                   6.50 usec
TE                  297.9 K
CNST6         125.0000000
CNST7         165.0000000
CNST13         10.0000000
CNST30          0.5981153
D0             0.00000300 sec
D1             1.43446398 sec
D6             0.05000000 sec
D16            0.00020000 sec
IN0            0.00001490 sec

======== CHANNEL f1 ========
SFO1       600.1327722 MHz
NUC1                    1H
P1                   10.60 usec
P2                   21.20 usec
PLW1           27.82500076 W

======== CHANNEL f2 ========
SFO2       150.9178738 MHz
NUC2                   13C
P3                    8.80 usec
P24                2000.00 usec
PLW2          78.13500214 W
SPNAM[7]     Crp60comp.4
SPOAL7               0.500
SPOFFS7  0 Hz
SPW7           9.24489975 W

====== GRADIENT CHANNEL =====
GPNAM[1]        SINE.100
GPNAM[3]        SINE.100
GPNAM[4]        SINE.100
GPNAM[5]        SINE.100
GPZ1               80.00 %
GPZ3               15.00 %
GPZ4              -10.00 %
GPZ5               -5.00 %
P16              1000.00 usec

F1 - Acquisition parameters
TD                     256
SFO1           150.9179 MHz
FIDRES       131.082214 Hz
SW              222.353 ppm
FnMODE     Echo-Antiecho

F2 - Processing parameters
SI                    2048
SF           600.1300654 MHz
WDW                   SINE
SSB       0
LB        0 Hz
GB        0
PC                    1.40

F1 - Processing parameters
SI                     512
MC2           echo-antiecho
SF           150.9028090 MHz
WDW                   SINE
SSB                      2
LB        0 Hz
GB        0
```

S7. COSY (600 MHz, $CDCl_3$) spectrum of derivative **1**

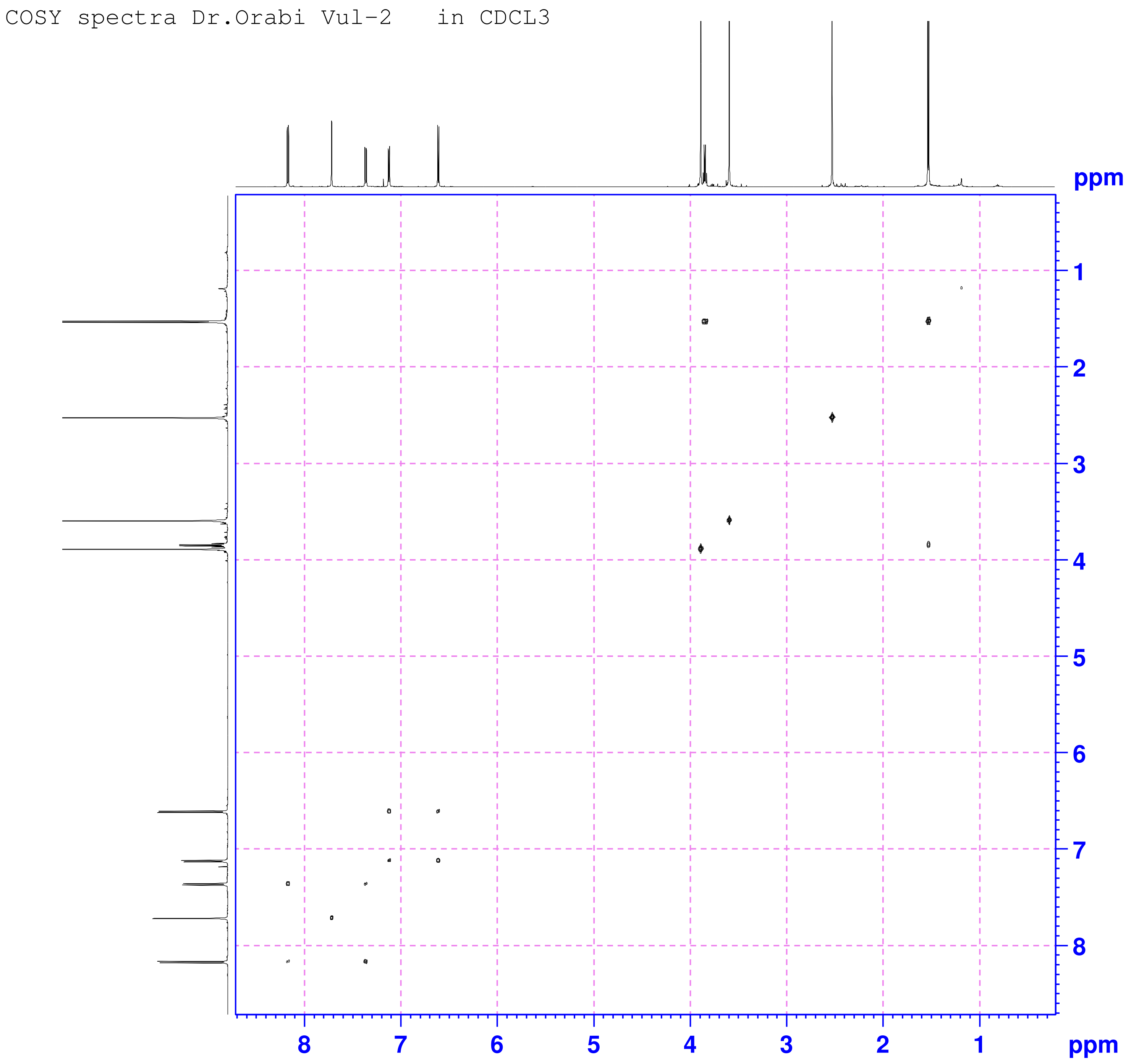


```
Current Data Parameters
NAME              Vul2-2D
EXPNO                   6
PROCNO                  1

F2 - Acquisition Parameters
Date_            20211025
Time                11.47
INSTRUM             spect
PROBHD   5 mm PABBO BB-
PULPROG       cosygpppqf
TD                   2048
SOLVENT             CDCl3
NS                      4
DS                      8
SWH              5102.041 Hz
FIDRES           2.491231 Hz
AQ              0.2007040 sec
RG                   90.5
DW                 98.000 usec
DE                   6.50 usec
TE                  298.0 K
D0             0.00000300 sec
D1             0.67709088 sec
D11            0.03000000 sec
D12            0.00002000 sec
D13            0.00000400 sec
D16            0.00020000 sec
IN0            0.00019600 sec

======== CHANNEL f1 ========
SFO1          600.1327447 MHz
NUC1                   1H
P0                  10.60 usec
P1                  10.60 usec
P17               2500.00 usec
PLW1          27.82500076 W
PLW10          5.00229979 W

====== GRADIENT CHANNEL =====
GPNAM[1]         SINE.100
GPZ1                20.00 %
P16               1000.00 usec

F1 - Acquisition parameters
TD                    320
SFO1             600.1327 MHz
FIDRES          15.943877 Hz
SW                  8.502 ppm
FnMODE                 QF

F2 - Processing parameters
SI                   2048
SF            600.1300658 MHz
WDW                  SINE
SSB       0
LB        0 Hz
GB        0
PC                   1.40

F1 - Processing parameters
SI                   2048
MC2                    QF
SF            600.1300653 MHz
WDW                  SINE
SSB       0
LB        0 Hz
GB        0
```

S8. ^{1}H NMR (600 MHz, $CDCl_3$) spectrum of derivative **2**

1H spectra Dr.Orabi Vul 3 in CDCL3

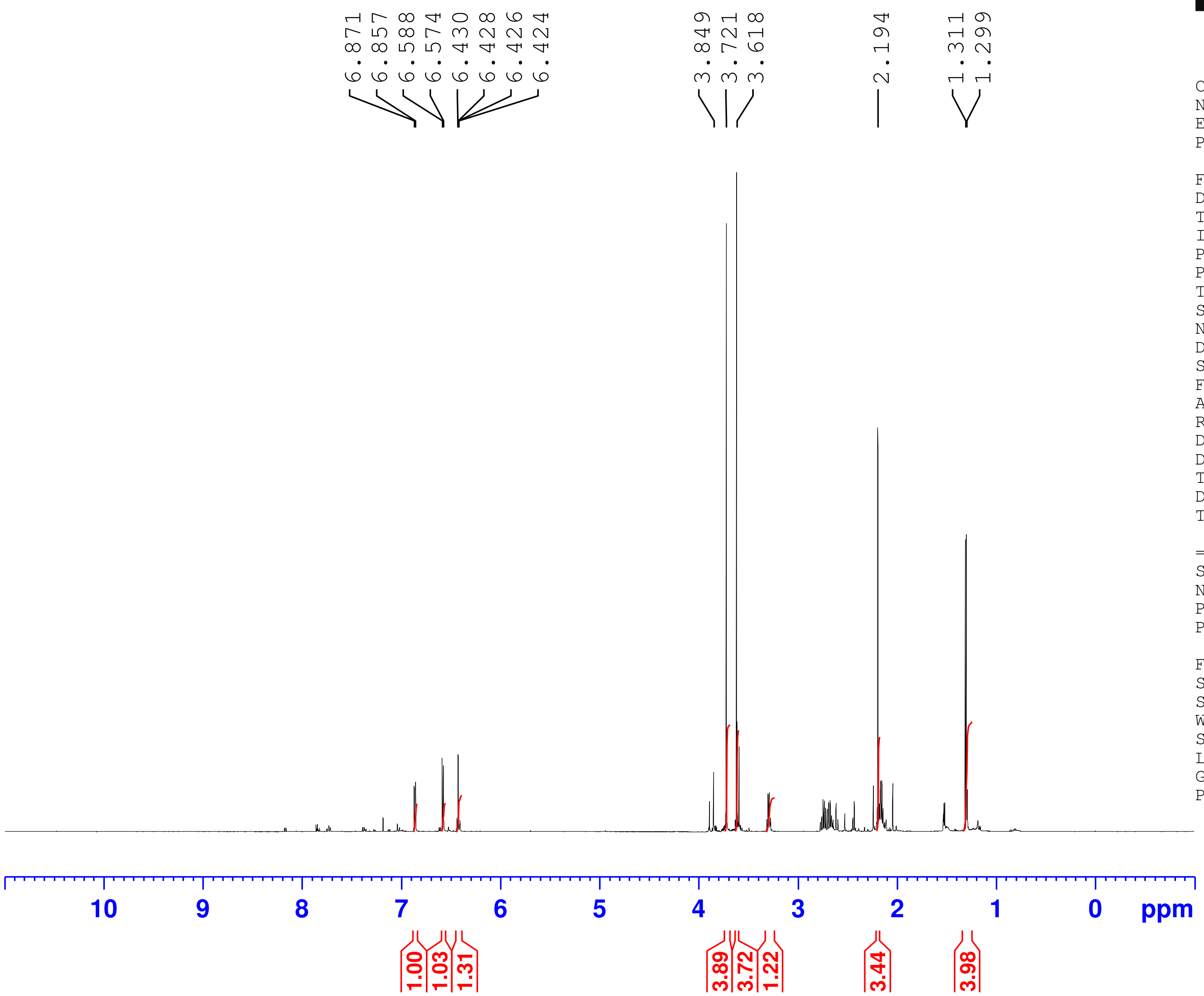

BRUKER

```
Current Data Parameters
NAME                Vul3-2D
EXPNO                     1
PROCNO                    1

F2 - Acquisition Parameters
Date_              20211025
Time                  14.42
INSTRUM               spect
PROBHD     5 mm PABBO BB-
PULPROG                zg30
TD                    65536
SOLVENT               CDCl3
NS                       16
DS                        2
SWH               12335.526 Hz
FIDRES             0.188225 Hz
AQ                2.6563926 sec
RG                      203
DW                   40.533 usec
DE                     6.50 usec
TE                    298.0 K
D1               1.00000000 sec
TD0                       1

======== CHANNEL f1 ========
SFO1            600.1337060 MHz
NUC1                     1H
P1                    10.60 usec
PLW1            27.82500076 W

F2 - Processing parameters
SI                    32768
SF              600.1300629 MHz
WDW                      EM
SSB      0
LB                     0.30 Hz
GB       0
PC                     1.00
```

S9. ^{13}C NMR (600 MHz, $CDCl_3$) spectrum of derivative **2**

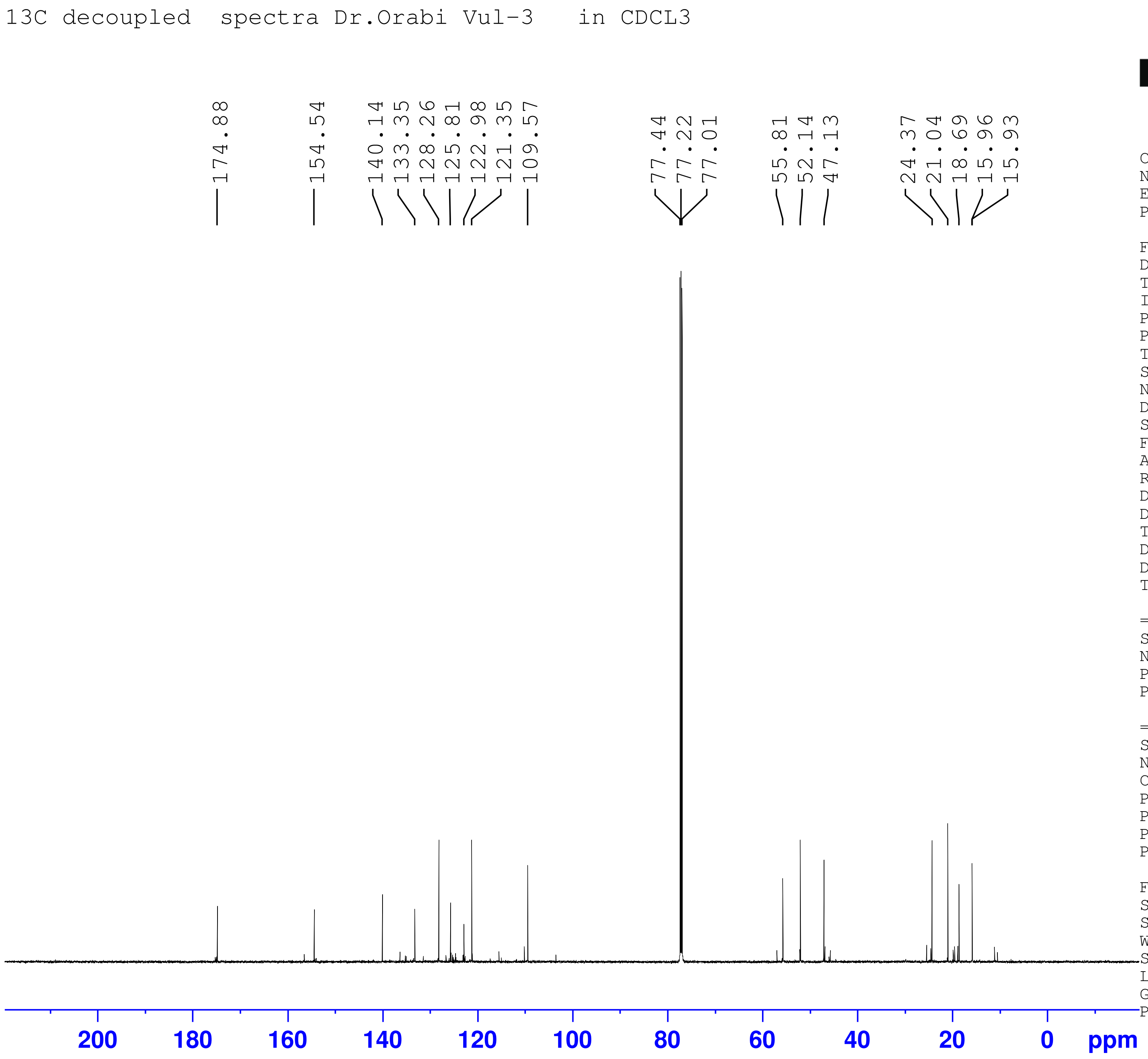

Current Data Parameters
NAME Vul3-2D
EXPNO 2
PROCNO 1

F2 - Acquisition Parameters
Date_ 20211025
Time 19.22
INSTRUM spect
PROBHD 5 mm PABBO BB-
PULPROG zgpg30
TD 65536
SOLVENT CDCl3
NS 5120
DS 4
SWH 36057.691 Hz
FIDRES 0.550197 Hz
AQ 0.9087659 sec
RG 203
DW 13.867 usec
DE 50.00 usec
TE 298.0 K
D1 2.00000000 sec
D11 0.03000000 sec
TD0 1

======== CHANNEL f1 ========
SFO1 150.9178979 MHz
NUC1 13C
P1 8.80 usec
PLW1 78.13500214 W

======== CHANNEL f2 ========
SFO2 600.1324005 MHz
NUC2 1H
CPDPRG[2 waltz65
PCPD2 70.00 usec
PLW2 27.82500076 W
PLW12 0.63804001 W
PLW13 0.32093000 W

F2 - Processing parameters
SI 32768
SF 150.9027795 MHz
WDW EM
SSB 0
LB 1.00 Hz
GB 0
PC 2.00

S10. DEPT-135 (600 MHz, $CDCl_3$) spectrum of derivative **2**

DEPT 135 spectra Dr.Orabi Vul-3 in CDCL3

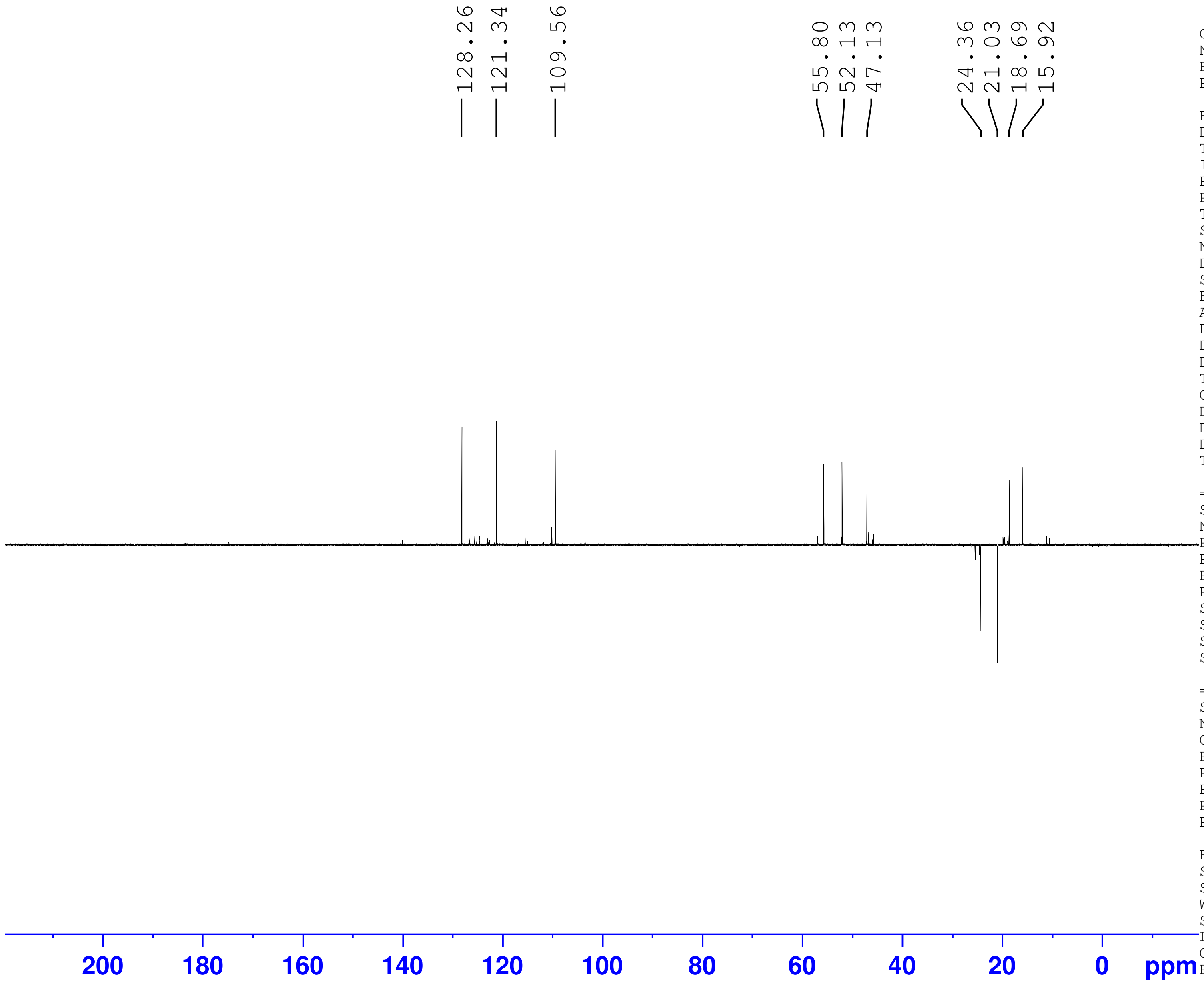


```
Current Data Parameters
NAME               Vul3-2D
EXPNO                    5
PROCNO                   1

F2 - Acquisition Parameters
Date_             20211026
Time                  0.34
INSTRUM              spect
PROBHD   5 mm PABBO BB-
PULPROG         deptsp135
TD                   65536
SOLVENT              CDCl3
NS                    2048
DS                       4
SWH             36057.691 Hz
FIDRES           0.550197 Hz
AQ              0.9087659 sec
RG                     203
DW                  13.867 usec
DE                   50.00 usec
TE                   298.0 K
CNST2          145.0000000
D1              2.00000000 sec
D2              0.00344828 sec
D12             0.00002000 sec
TD0                      1

======== CHANNEL f1 ========
SFO1           150.9178979 MHz
NUC1                   13C
P1                    8.80 usec
P13                2000.00 usec
PLW0     0 W
PLW1           78.13500214 W
SPNAM[5]       Crp60comp.4
SPOAL5               0.500
SPOFFS5  0 Hz
SPW5            9.24489975 W

======== CHANNEL f2 ========
SFO2           600.1324005 MHz
NUC2                    1H
CPDPRG[2           waltz65
P3                   10.60 usec
P4                   21.20 usec
PCPD2                70.00 usec
PLW2           27.82500076 W
PLW12           0.63804001 W

F2 - Processing parameters
SI                   32768
SF             150.9027805 MHz
WDW                     EM
SSB      0
LB                    1.00 Hz
GB       0
PC                    3.00
```

S11. HSQC (600 MHz, $CDCl_3$) spectrum of derivative **2**

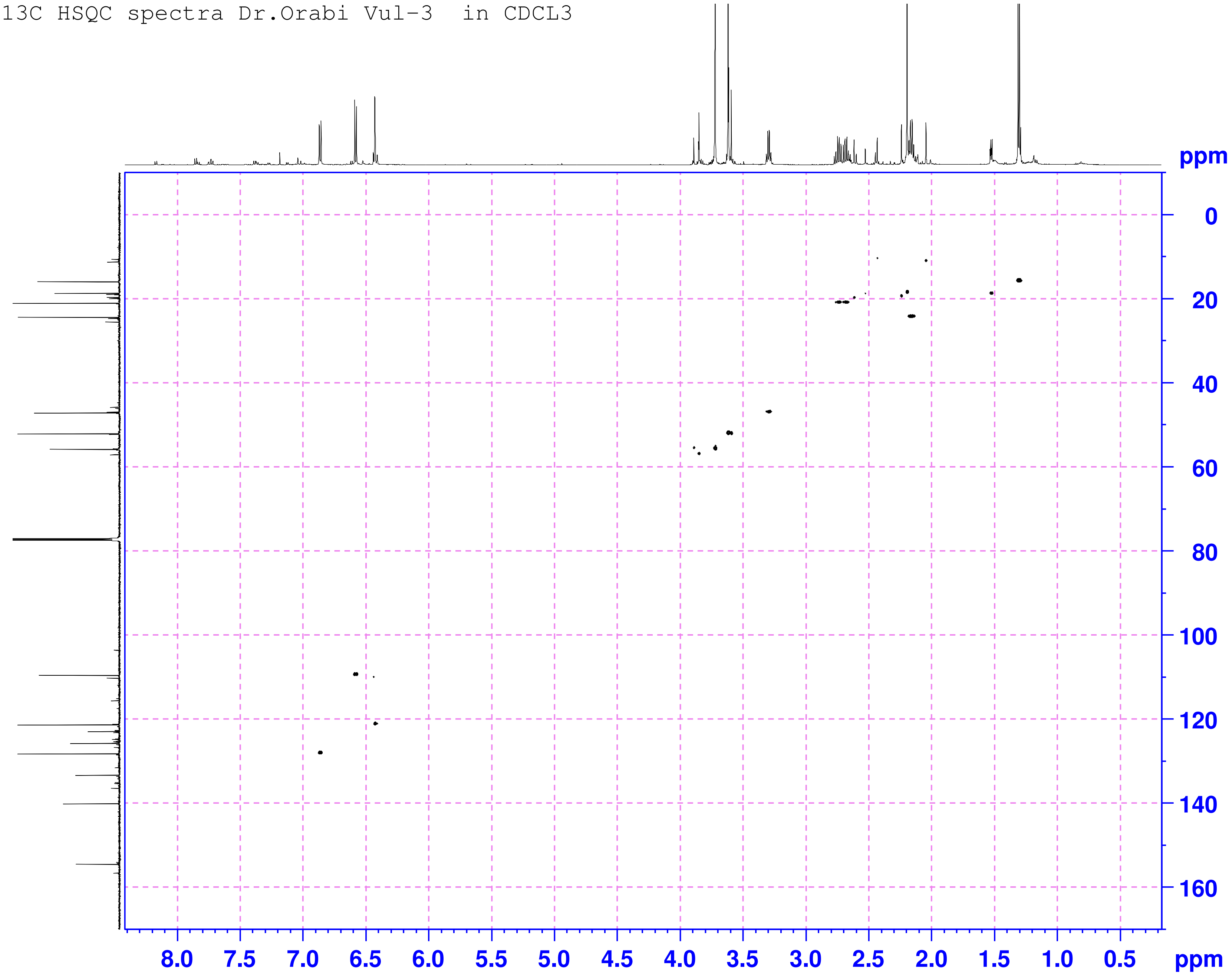

BRUKER

```
Current Data Parameters
NAME              Vul3-2D
EXPNO                   7
PROCNO                  1

F2 - Acquisition Parameters
Date_            20211026
Time                 1.01
INSTRUM             spect
PROBHD    5 mm PABBO BB-
PULPROG    hsqcetgpsp.2
TD                   2048
SOLVENT             CDCl3
NS                      8
DS                     16
SWH              4950.495 Hz
FIDRES           2.417234 Hz
AQ              0.2068480 sec
RG                    203
DW                101.000 usec
DE                   6.50 usec
TE                  298.1 K
CNST2         145.0000000
D0             0.00000300 sec
D1             1.89964795 sec
D4             0.00172414 sec
D11            0.03000000 sec
D16            0.00020000 sec
IN0            0.00001840 sec
ZGOPTNS

======== CHANNEL f1 ========
SFO1          600.1326395 MHz
NUC1                   1H
P1                  10.60 usec
P2                  21.20 usec
P28               1000.00 usec
PLW1          27.82500076 W

======== CHANNEL f2 ========
SFO2          150.9148803 MHz
NUC2                  13C
CPDPRG[2             garp4
P3                   8.80 usec
P14                500.00 usec
P24               2000.00 usec
PCPD2               60.00 usec
PLW0     0 W
PLW2          78.13500214 W
PLW12          1.68079996 W
SPNAM[3] Crp60,0.5,20.1
SPOAL3              0.500
SPOFFS3  0 Hz
SPW3           9.24489975 W
SPNAM[7]     Crp60comp.4
SPOAL7              0.500
SPOFFS7  0 Hz
SPW7           9.24489975 W

====== GRADIENT CHANNEL =====
GPNAM[1]         SINE.100
GPNAM[2]         SINE.100
GPZ1                80.00 %
GPZ2                20.10 %
P16               1000.00 usec

F1 - Acquisition parameters
TD                    512
SFO1             150.9149 MHz
FIDRES          53.074047 Hz
SW                180.061 ppm
FnMODE       Echo-Antiecho

F2 - Processing parameters
SI                   2048
SF            600.1300629 MHz
WDW                 QSINE
SSB                     2
LB       0 Hz
GB       0
PC                   1.40

F1 - Processing parameters
SI                   1024
MC2          echo-antiecho
SF            150.9028090 MHz
WDW                 QSINE
SSB                     2
LB       0 Hz
GB       0
```

S12. HMBC (600 MHz, $CDCl_3$) spectrum of derivative **2**

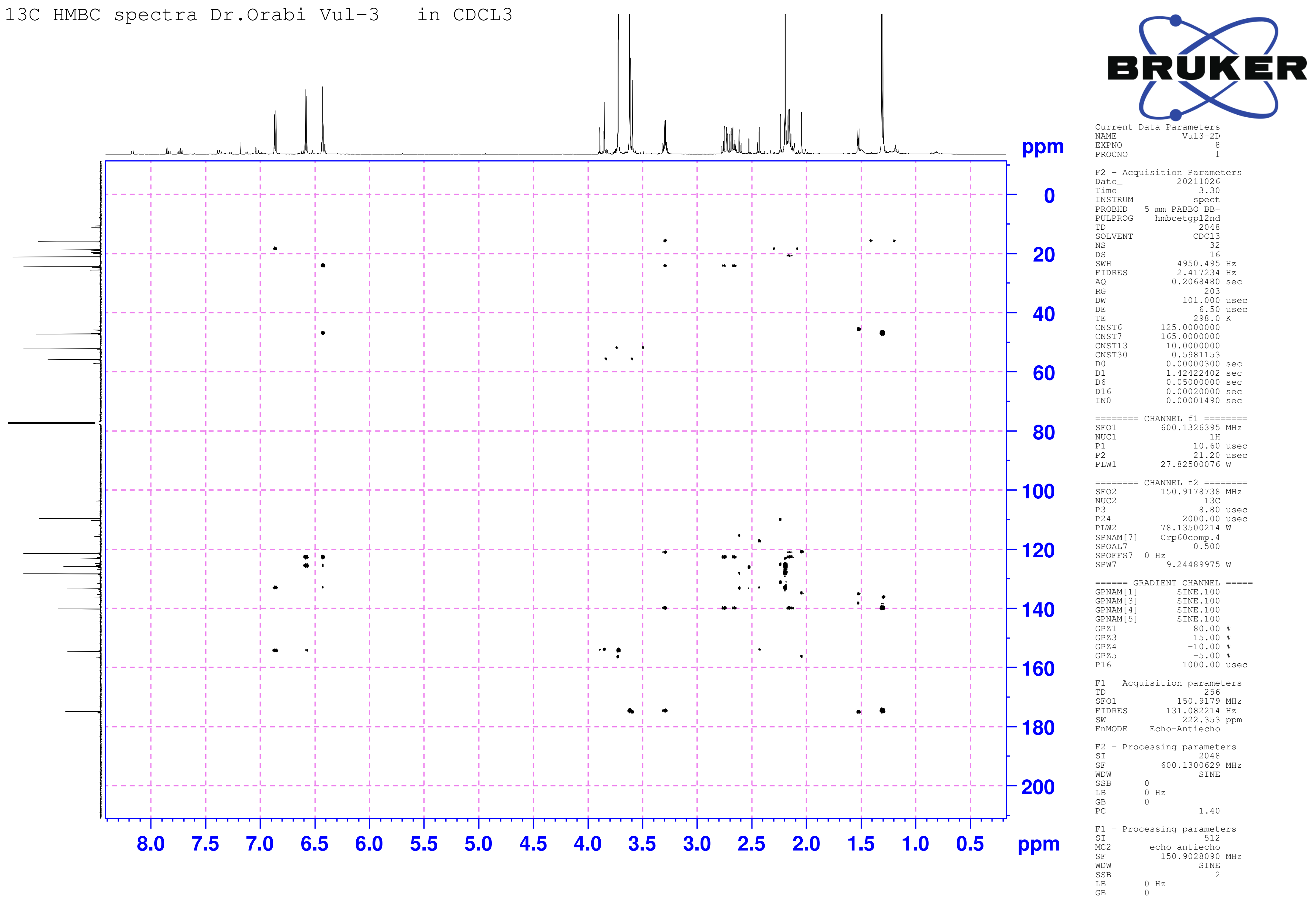

S13. HMBC (600 MHz, $CDCl_3$) spectrum of derivative **2**

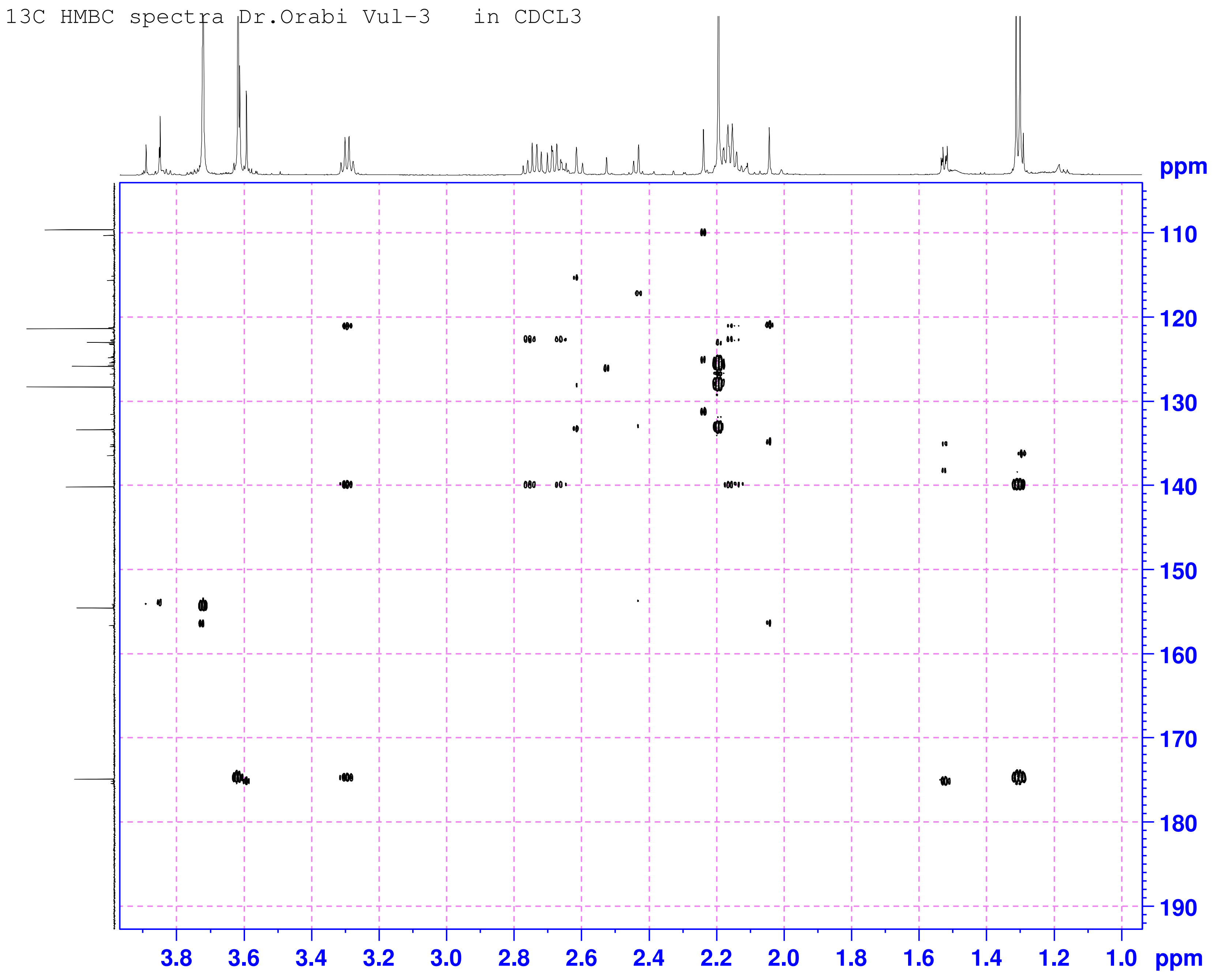


```
Current Data Parameters
NAME              Vul3-2D
EXPNO                   8
PROCNO                  1

F2 - Acquisition Parameters
Date_            20211026
Time                 3.30
INSTRUM             spect
PROBHD    5 mm PABBO BB-
PULPROG     hmbcetgpl2nd
TD                   2048
SOLVENT             CDCl3
NS                     32
DS                     16
SWH              4950.495 Hz
FIDRES           2.417234 Hz
AQ              0.2068480 sec
RG                    203
DW                101.000 usec
DE                   6.50 usec
TE                  298.0 K
CNST6         125.0000000
CNST7         165.0000000
CNST13         10.0000000
CNST30          0.5981153
D0             0.00000300 sec
D1             1.42422402 sec
D6             0.05000000 sec
D16            0.00020000 sec
IN0            0.00001490 sec

======== CHANNEL f1 ========
SFO1          600.1326395 MHz
NUC1                   1H
P1                  10.60 usec
P2                  21.20 usec
PLW1          27.82500076 W

======== CHANNEL f2 ========
SFO2          150.9178738 MHz
NUC2                  13C
P3                   8.80 usec
P24               2000.00 usec
PLW2          78.13500214 W
SPNAM[7]      Crp60comp.4
SPOAL7              0.500
SPOFFS7   0 Hz
SPW7           9.24489975 W

====== GRADIENT CHANNEL =====
GPNAM[1]         SINE.100
GPNAM[3]         SINE.100
GPNAM[4]         SINE.100
GPNAM[5]         SINE.100
GPZ1                80.00 %
GPZ3                15.00 %
GPZ4               -10.00 %
GPZ5                -5.00 %
P16               1000.00 usec

F1 - Acquisition parameters
TD                    256
SFO1             150.9179 MHz
FIDRES         131.082214 Hz
SW                222.353 ppm
FnMODE       Echo-Antiecho

F2 - Processing parameters
SI                   2048
SF            600.1300629 MHz
WDW                  SINE
SSB       0
LB        0 Hz
GB        0
PC                   1.40

F1 - Processing parameters
SI                    512
MC2           echo-antiecho
SF            150.9028090 MHz
WDW                  SINE
SSB                     2
LB        0 Hz
GB        0
```

S14. COSY (600 MHz, $CDCl_3$) spectrum of derivative **2**

COSY spectra Dr.Orabi Vul-3   in CDCL3

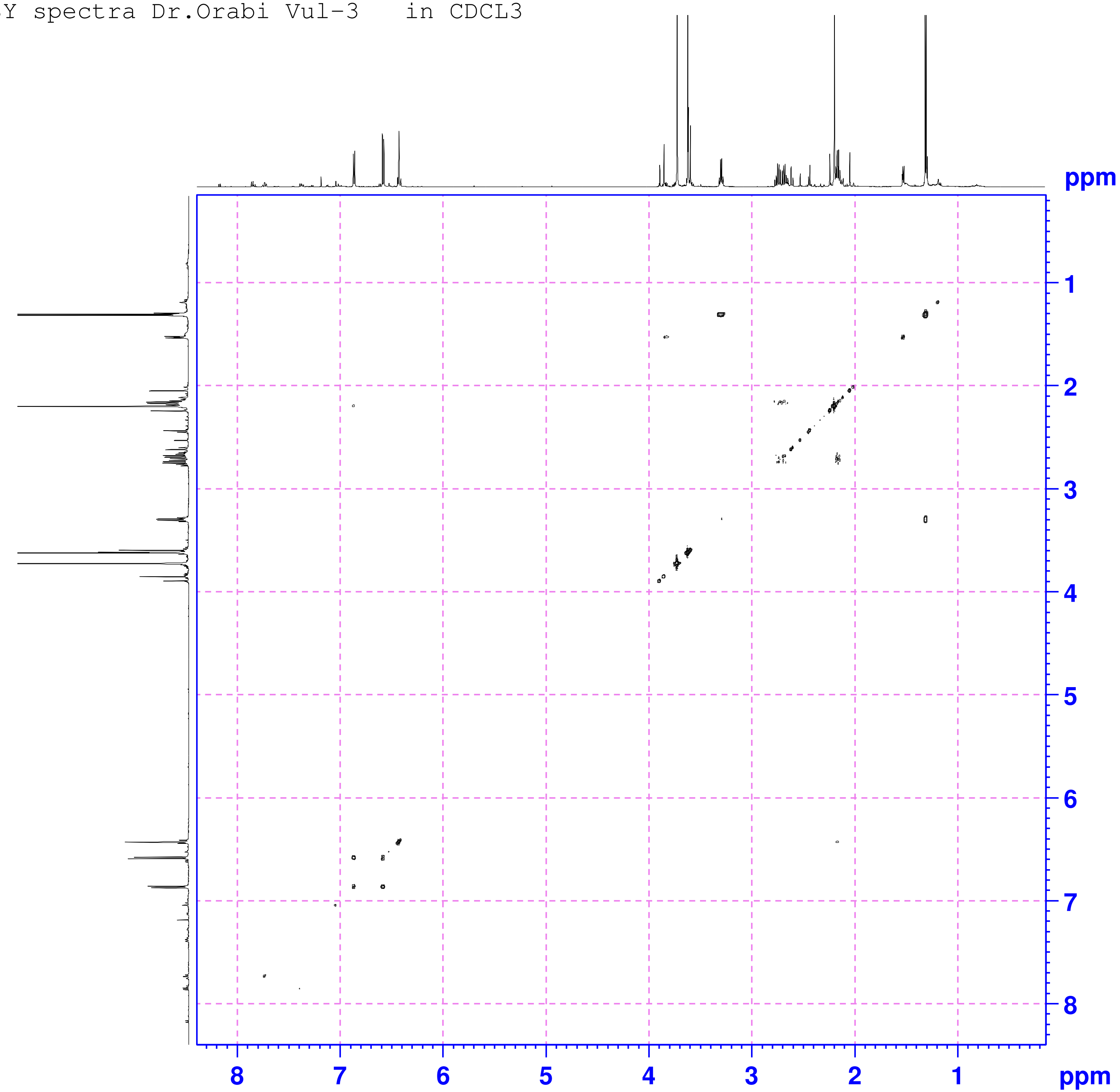


```
Current Data Parameters
NAME              Vul3-2D
EXPNO                   6
PROCNO                  1

F2 - Acquisition Parameters
Date_            20211026
Time                 0.36
INSTRUM             spect
PROBHD   5 mm PABBO BB-
PULPROG        cosygpppqf
TD                   2048
SOLVENT             CDCl3
NS                      4
DS                      8
SWH              4950.495 Hz
FIDRES           2.417234 Hz
AQ              0.2068480 sec
RG                   90.5
DW                101.000 usec
DE                   6.50 usec
TE                  297.9 K
D0             0.00000300 sec
D1             0.67094702 sec
D11            0.03000000 sec
D12            0.00002000 sec
D13            0.00000400 sec
D16            0.00020000 sec
IN0            0.00020200 sec

======== CHANNEL f1 ========
SFO1          600.1326237 MHz
NUC1                   1H
P0                  10.60 usec
P1                  10.60 usec
P17               2500.00 usec
PLW1          27.82500076 W
PLW10          5.00229979 W

====== GRADIENT CHANNEL =====
GPNAM[1]         SINE.100
GPZ1                20.00 %
P16               1000.00 usec

F1 - Acquisition parameters
TD                    320
SFO1             600.1326 MHz
FIDRES          15.470297 Hz
SW                  8.249 ppm
FnMODE                 QF

F2 - Processing parameters
SI                   2048
SF            600.1300618 MHz
WDW                  SINE
SSB      0
LB       0 Hz
GB       0
PC                   1.40

F1 - Processing parameters
SI                   2048
MC2                    QF
SF            600.1300594 MHz
WDW                  SINE
SSB      0
LB       0 Hz
GB       0
```
